# Modelling and robust controller design for an underactuated self-balancing robot with uncertain parameter estimation

**DOI:** 10.1371/journal.pone.0285495

**Published:** 2023-08-09

**Authors:** Osama A. Choudhry, Muhammad Wasim, Ahsan Ali, Mohammad Ahmad Choudhry, Jamshed Iqbal

**Affiliations:** 1 Department of Physics and Electrical Engineering, Universität Bremen, Bremen, Germany; 2 Department of Aeronautics and Astronautics Engineering, Institute of Space Technology, Islamabad, Pakistan; 3 Department of Electrical Engineering, University of Engineering and Technology, Taxila, Pakistan; 4 School of Computer Science, Faculty of Science and Engineering, University of Hull, Hull, United Kingdom; Zonguldak Bülent Ecevit University: Zonguldak Bulent Ecevit Universitesi, TURKEY

## Abstract

A comprehensive literature review of self-balancing robot (SBR) provides an insight to the strengths and limitations of the available control techniques for different applications. Most of the researchers have not included the payload and its variations in their investigations. To address this problem comprehensively, it was realized that a rigorous mathematical model of the SBR will help to design an effective control for the targeted system. A robust control for a two-wheeled SBR with unknown payload parameters is considered in these investigations. Although, its mechanical design has the advantage of additional maneuverability, however, the robot’s stability is affected by changes in the rider’s mass and height, which affect the robot’s center of gravity (COG). Conventionally, variations in these parameters impact the performance of the controller that are designed with the assumption to operate under nominal values of the rider’s mass and height. The proposed solution includes an extended Kalman filter (EKF) based sliding mode controller (SMC) with an extensive mathematical model describing the dynamics of the robot itself and the payload. The rider’s mass and height are estimated using EKF and this information is used to improve the control of SBR. Significance of the proposed method is demonstrated by comparing simulation results with the conventional SMC under different scenarios as well as with other techniques in literature. The proposed method shows zero steady state error and no overshoot. Performance of the conventional SMC is improved with controller parameter estimation. Moreover, the stability issue in the reaching phase of the controller is also solved with the availability of parameter estimates. The proposed method is suitable for a wide range of indoor applications with no disturbance. This investigation provides a comprehensive comparison of available techniques to contextualize the proposed method within the scope of self-balancing robots for indoor applications.

## Introduction

With growing size of indoor commercial facilities like airports, warehouses, hospitals, and shopping malls need for two-wheel self-balancing robots having provision for the rider is growing. This is a compelling need for social service providers and operation personals to provide state of the art services to the public. Unfortunately, pace of research in this area is slow. A safe and efficient two-wheel SBR is expected to enhance the performance of the indoor commercial facilities.

A reasonable accurate mathematical model of two-wheel self-balancing robot (SBR) including rider and equipped with a controller capable of disturbance rejection and handling uncertainties have to be developed. Suitable parameter estimation approach will make available unknow data to the control system without installing costly measuring sensors and actuators on the robot. A suitable estimation algorithm integrated with the nonlinear controller will make it possible to develop a cost effective and efficient SBR with provision of rider.

Unfortunately, the available solution uses expensive measuring systems to measure unknow parameters or have no rider facility. Best existing approach uses gyroscope and inertial measuring units to measure unknow data and make the solution expensive and cumbersome. We hope to integrate Extended Kalman Filter with Sliding mode controller to simplify the SBR with rider and make it efficient for indoor applications.

Due to theoretical significance of the inverted pendulum, it has been investigated as one of the most fundamental problems in control system theory. It was used time and again to comprehend experimental models, corroborate the efficiency of emerging control techniques, and substantiate their implementations [1, 2]. One of its practical applications included a self-balancing two-wheeled transport system or a self-balancing robot (SBR). These robots have faster maneuverability which makes them very useful for several applied areas. These systems are a modified form of a simple cart and pendulum system, with wheels located at multiple positions. Modeling and control design of the cart and pendulum system laid the foundation for the required SBR algorithms [3, 4]. In literature, fabricated SBR systems range from scaled prototypes to life-size robots. Researchers have carried out extensive modeling of the robot system using Euler Lagrange equations and Newtonian dynamic modeling method. Various control techniques were utilized in the design and implementation of upright stability control for these robots [5–8].

Different types of self-balancing robots have been used for multiple applications and environments. This calls for a substantial dynamic model of a nonholonomic SBR. Although this problem was investigated earlier, however, the mathematical model encapsulated the dynamics of a riderless prototype with a sluggish control response [9]. As a tradeoff, some nonlinear and perceptual control techniques were used to incorporate robustness against system uncertainties of riderless SBRs. Nonetheless, the disturbance rejection capability of the control system was sluggish and the tilt response had significant oscillations [10, 11]. Altan and Hacıoglu have used three-axis gimble system mounted on UAV for external disturbance rejection to solve target tracking problem. However, this system may not be feasible to install on SBR system due to its nature of application and cost [12]. Hence, to delineate an exact SBR mathematically, it is important to consider the variations in payload parameters of the system that cause a mismatch in the model and the actual robot dynamics. This mismatch occurs due to error in the global center of mass and moment of inertia of the system. This can ultimately affect the disturbance rejection capability of the controller [13–15]. Although more robust control algorithms have been developed for system stability, however, they allow payload variations within a narrow upper and lower bound [16–19].

SMC design can be broadly categorized into two parts. The first part deals with the designing of a sliding surface with desirable attributes like tracking and stability. The second part entails the designing of discontinuous control law that makes the sliding surface an invariant set. This control law also ensures that the sliding surface has finite time reachability. Some researchers used fuzzy based SMC to solve the control system problems. For low level of external disturbances, the chattering problem in SMC was addressed but for higher disturbances level chattering effects were more pronounced [20–22]. Kim and Kwon have suggested disturbance compensation method for two-wheel robot. They have solved the problem of disturbance rejection effectively for the robot without rider on it [23]. In another work, effectiveness of adaptive self-balancing technique has been illustrated by using payloads of 85 kg and 60 kg. Nonetheless, this research did not discuss variations outside this range of payload with a linear model to design and realize the stability control [24]. Arbitrary Order Sliding Mode Control was another approach that **was** used to mitigate disturbances in static systems. This approach is bound to fail for dynamic systems with payload variations [25].

In another proposal, a multi-objective controller design to tackle the payload uncertainties for an electric unicycle was investigated. Even though, it takes into account the variations in the swing arm of the vehicle, however, the simulations lack the validation of the rider’s mass variation [26]. A self-balancing robot, KUWAY, was developed at Kookmin University. This robot used a control moment gyroscope that effectively rejects disturbances caused due to the change in the payload without changing its position. However, this method requires a lot of space on the robotic system. Also, it is noisy, expensive, and had poor power efficiency [27]. Another proposition was made by researchers at Pusan National University, Busan, Korea. They incorporated series elastic actuators into the base of SBR that credibly compensates for rider inertia during acceleration and deceleration. While the results were substantial, mechanical impedance was generated by the actuators and the hardware adds to the expense of the final product [28]. Payload dynamics can be separately incorporated into the system. Some researchers modeled the mass, height, and moment of inertia of payload separately from the robot mass, height, and moment of inertia. The mass in the human body is not uniformly distributed. Hence, the COG in the case of a rider is defined to be a bit higher than the geometric center [29, 30]. However, this does not enable the system to accommodate any change in the payload. The controller designed would still have a limited payload allowance unless an extra sensor is used to actively measure the change in mass and height of the rider.

Several SBRs have taken into consideration the payload variations and have provided some solutions for them. Two self-balancing robot systems, with one having a linear workspace extension, have been developed at the Chungnam National University. A simple self-balancing robot system used a neuro-fuzzy control algorithm to reject load disturbances. However, the magnitude of the load disturbance was very small as compared to the disturbances caused by a dynamic rider. The robot with linear workspace extension devised control strategies for a platform that can extend itself linearly with mass being constant [31, 32]. A material handling platform has also been developed by Louwrens J. Butler and Glen Bright. The material handling platform used an LQR controller to stabilize itself, but the deviation in load parameters degraded the control performance. For example, in this case, the controller requires approximately 7 seconds to come back to its reference angle after disturbance rejection [33].

Advanced techniques have been developed to reconstruct the dynamics of the uncertain Euler–Lagrange systems. This includes the optimal bounded ellipsoid (OBE) identification which uses upper and lower bounds to prevent blow-up and ensure good identification performance for time-varying parameters [34]. Whale and Grasshopper optimization algorithms-based adaptive control systems have been used for 3D printer to efficiently reject disturbances. However, they are limited to PID controllers and fail to handle parameter variations of SBR with rider dynamics [35, 36]. Jasim has used grey wolf optimization-based State feedback controller for two-wheeled self-balancing robot. Neither he has considered payload in SBR nor external disturbances [37].

Another way of looking at the problem is to link unknown parameters to the known parameters. For instance, variation in mass and height of the payload can generate a pitch angle difference between the geometrical center of the robot and the gravity center of the combined body. Perturbations caused due to this angle can be adjusted using control algorithms [33]. To further increase the effectiveness of the controller, an observer can be designed to identify and compensate for modeling mismatch and add robustness to the control strategy [38]. Although, this is a very effective way to deal with the modeling mismatch, however, these approaches cannot be used in the case of a personal transporter. A controller has been designed and validated for a two-wheeled mobile manipulator whose application and dynamics slightly vary from that of an SBR. Furthermore, the observer estimates the modeling mismatches of the robot mass and manipulator arm length, leaving the parameters of payload undetermined [39]. To deal with modeling uncertainties, a robust control strategy can be devised with adaptive simultaneous stabilization of multiple degrees-of-freedom robot systems. Although the technique looked promising, the varying control gain caused the initial control torque to shoot-up considerably under non-nominal conditions. This can originate problems, considering the saturation limit associated with electric motors [40]. Chand et al investigated Certainty equivalence-based robust sliding mode control for disturbance for maximum power extraction from wind turbine. However, for a reasonable performances an estimation technique is required to integrated with the suggested approach [41]. The rider’s height and mass, being variable parameters, may cause a mismatch in the real model of the system by changing moment of inertia and shifting the position of COG of the combined system. Hence, these parameters need to be added to the mathematical model. The rider’s mass and height were unknown and cannot be measured easily. A feasible solution was required to address the parameter estimation problem. A generalized estimator has been designed using an EKF that worked in combination with SMC. EKF and its variants has been utilized in literature for the parameter estimation in many systems. It has been used for the aerodynamic model estimation and model uncertainty estimation of airship [42–44]. EKF can estimate the mismatch caused by the varying payload parameters. The knowledge of this mismatch has been utilized to minimize the gain of the SMC. Due to phase delays in the system, these low gains have been verified to reduce the initial magnitude and oscillations in the control effort. Therefore, maximizing system performance with a minimum control effort requirement.

### Major contributions and novelty: -

- Derivation of the mathematical model of the SBR with the inclusion of variable payload dynamics and their effect on the system states.
- Inclusion of motor parameters with decoupled voltage signals as the control input.
- Design of sliding mode controller for the proposed mathematical model.
- Design of an EKF based parameter estimator.
- Proposed ESMC technique and its comparison with the existing SMC stability method.
- Validation of the results using simulations.
- Comparison of the results with existing techniques to justify novelty.

Section 2 describes the detailed mathematical model for a life-size SBR capable of transporting a human rider. Newtonian modeling approach is used to develop an elaborated model. Through the model equations an insight into the dynamics of SBR is developed. In section 3, Designed of a nonlinear sliding mode control system to regulate the pitch and yaw angle of the SBR is discussed. Sliding surface design needs the information of modeling error. Estimation of error is made through extended Kalman filter and used to design the sliding mode controller. Section 4, investigates the proposed algorithm for EKF based SMC to ensure stability of SBR. The proposed estimation algorithm requires carefully tune the EKF parameters to achieve the required performance. In section 5, controller stability analysis is performed using the SBR model, the sliding surfaces defined, given controllers, and the EKF estimator designed for estimation of ${\hat{M}}_{R}$ and ${\hat{L}}_{r}$. Section 6 covers the discussions on simulations conducted to validate the proposed ESMC balancing control. The objective of these simulations is to demonstrate the enhanced performance of the proposed method on account of the estimations made for ${\hat{M}}_{R}$ and ${\hat{L}}_{r}$. and section 7 concludes the investigations with emphasizing major achievements of the proposed algorithms in comparison with existing works in the literature. It also gives a future road map to further research in this field for interested researchers.

### Description and modeling of self-balancing robot

**Fig 1:**
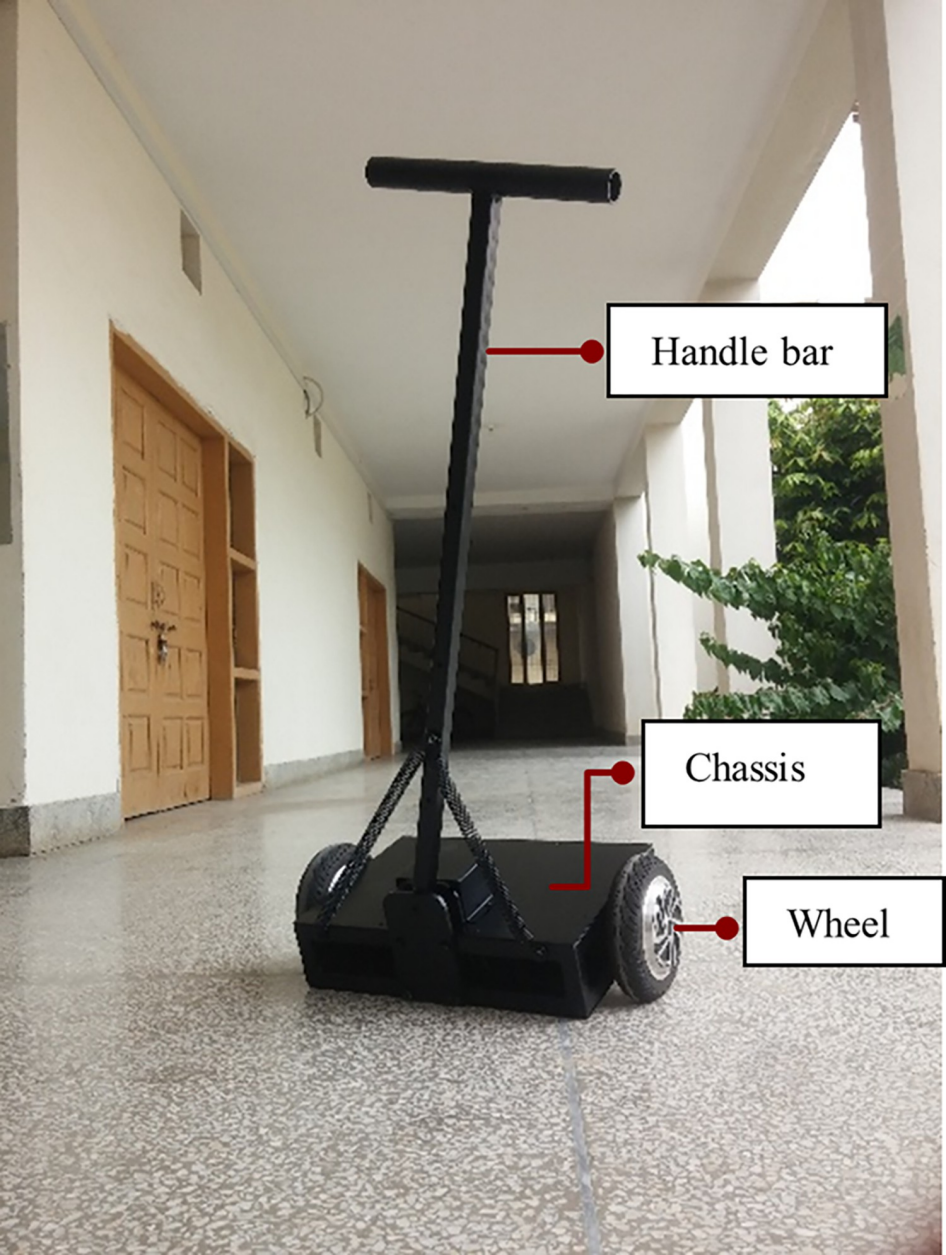
Self-balancing robot (SBR) in parking position.

Fig 1 shows a life-size SBR capable of transporting a human rider which is the system under consideration in this research. The robot dimensions are given in the nomenclature section.

**Fig 2:**
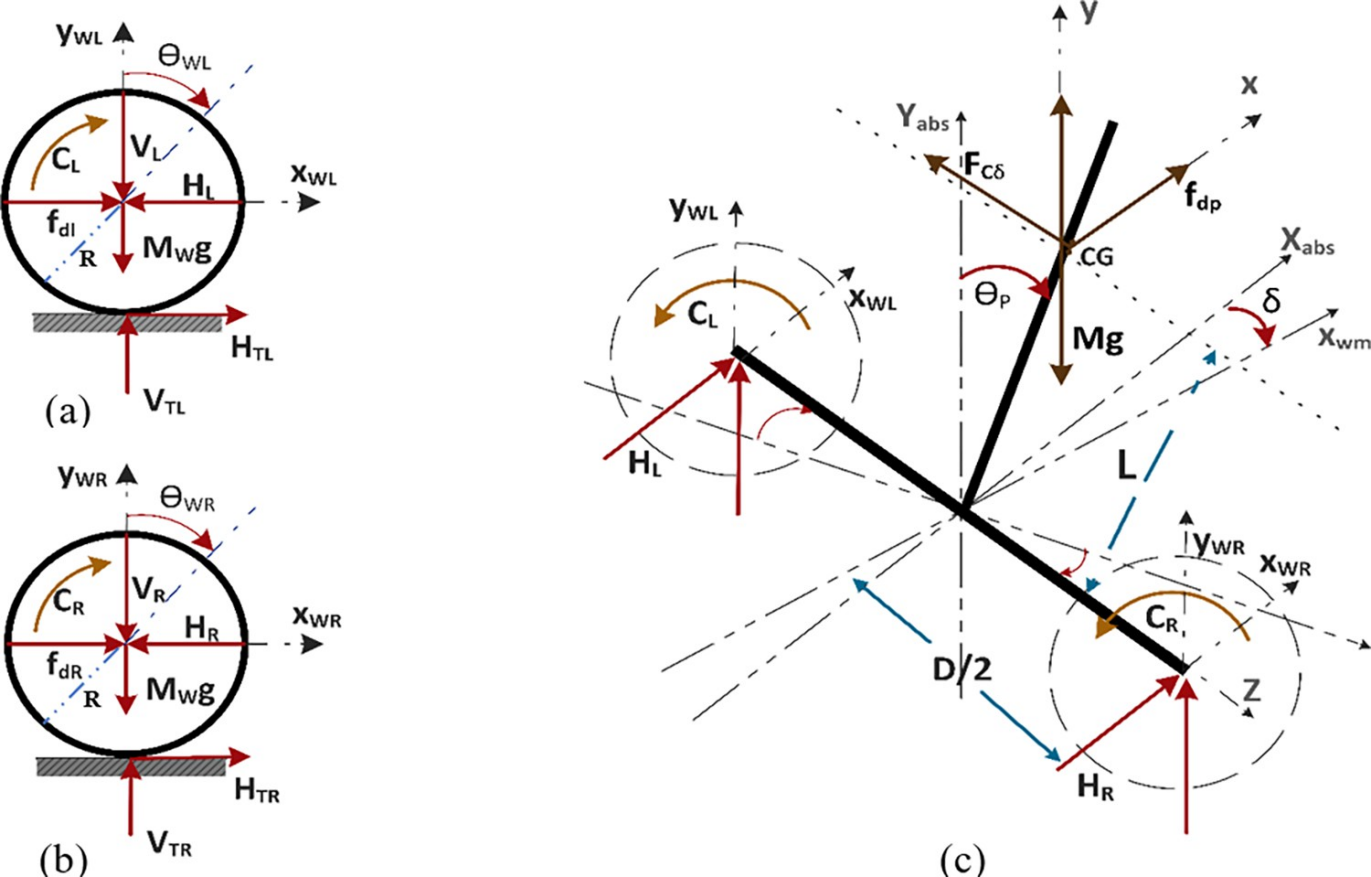
Free body diagram of the self-balancing robot platform (a) left wheel (b) right wheel (c) chassis.

The actuation and stability of the robot are attained through a feedback control mechanism. An inertial measurement unit(IMU) is embedded in the center of robot’s chassis. As the rider is tilted forward, pitch and yaw rate data measured through IMU is fed back to the controller. This information is processed by the controller and generates a control torque command to adjust the states of the robot accordingly. Hence, performing stabilization and actuation action on the SBR, simultaneously. To design an effective control system, the dynamics of the physical body must be modeled mathematically. The model used in this paper is based on the parameters characterizing the robot. Model equations are resulting equations are derived using Newtonian modeling approach. Fig 2 shows the free body diagram (FBD) of the SBR used for the derivation of a mathematical model in this investigation. The FBD is divided into sub-diagrams to effectively extract the equations that represent the dynamics of the system. As the Newtonian method is used to model the system, linear and angular versions of the second law of motion is used to describe the sum of forces and torques, respectively.


$$\alpha =\frac{\sum T}{I}$$ (1)

∑F=ma (2)

**Fig 3:**
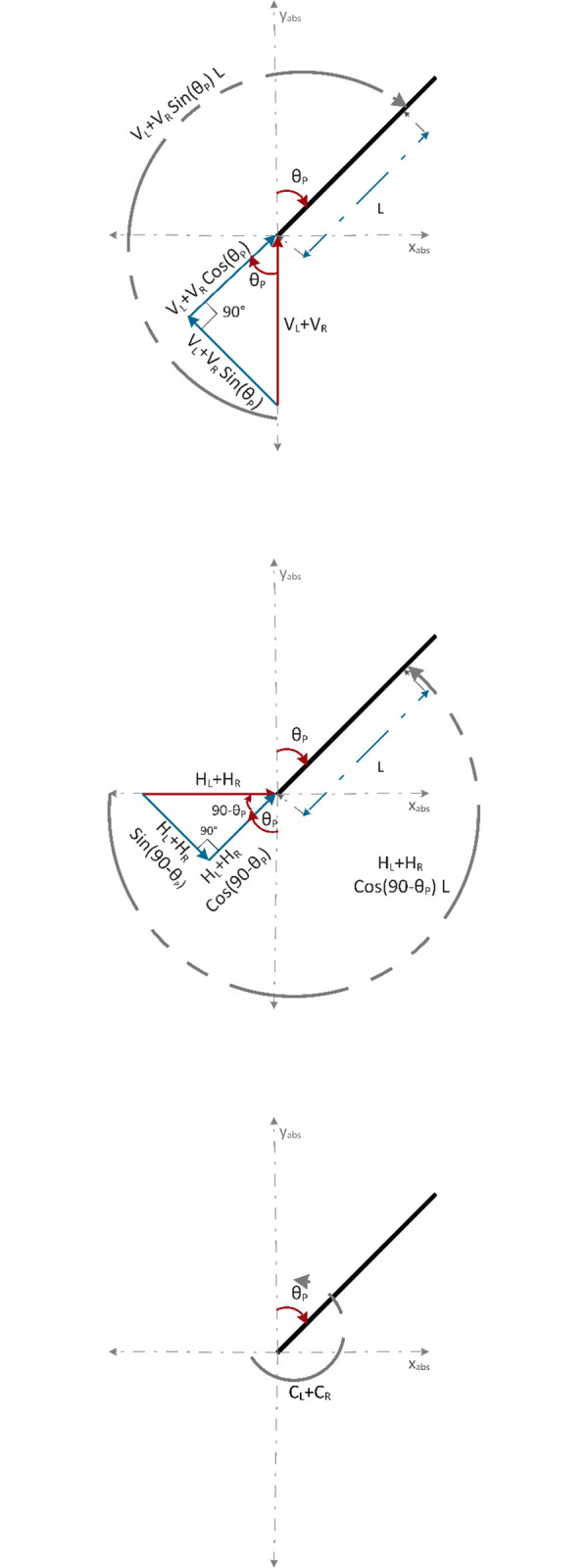
Torque diagram about the z-axis (a) vertical reaction forces between wheels and chassis (b) horizontal reaction forces between wheels and chassis, (c) control torques from left and right wheel.

Fig 3 shows the sum of torques observed about the z-axis. As a convention for derivation, clockwise torque is taken as positive and counterclockwise torque as negative. Sin *θ*_*P*_ component of (*V*_*L*_+*V*_*R*_) and sin (90−*θ*_*P*_) component of (*H*_*L*_+*H*_*R*_) generate the required torques. Since, sin (90−*θ*_*P*_) = cos *θ*_*P*_, both *H*_*L*_+*H*_*R*_ and *C*_*L*_+*C*_*R*_ generate counterclockwise torques and (*V*_*L*_+*V*_*R*_) contribute to the clockwise torque. Eq *(3)* represents the sum of torques observed about the z-axis.


$$J{\ddot{\theta }}_{P}=(V_{L}+V_{R})L\;\sin\;\theta_{P}-(H_{L}+H_{R})L\;\cos\;\theta_{P}-(C_{L}+C_{R})$$ (3)

**Fig 4:**
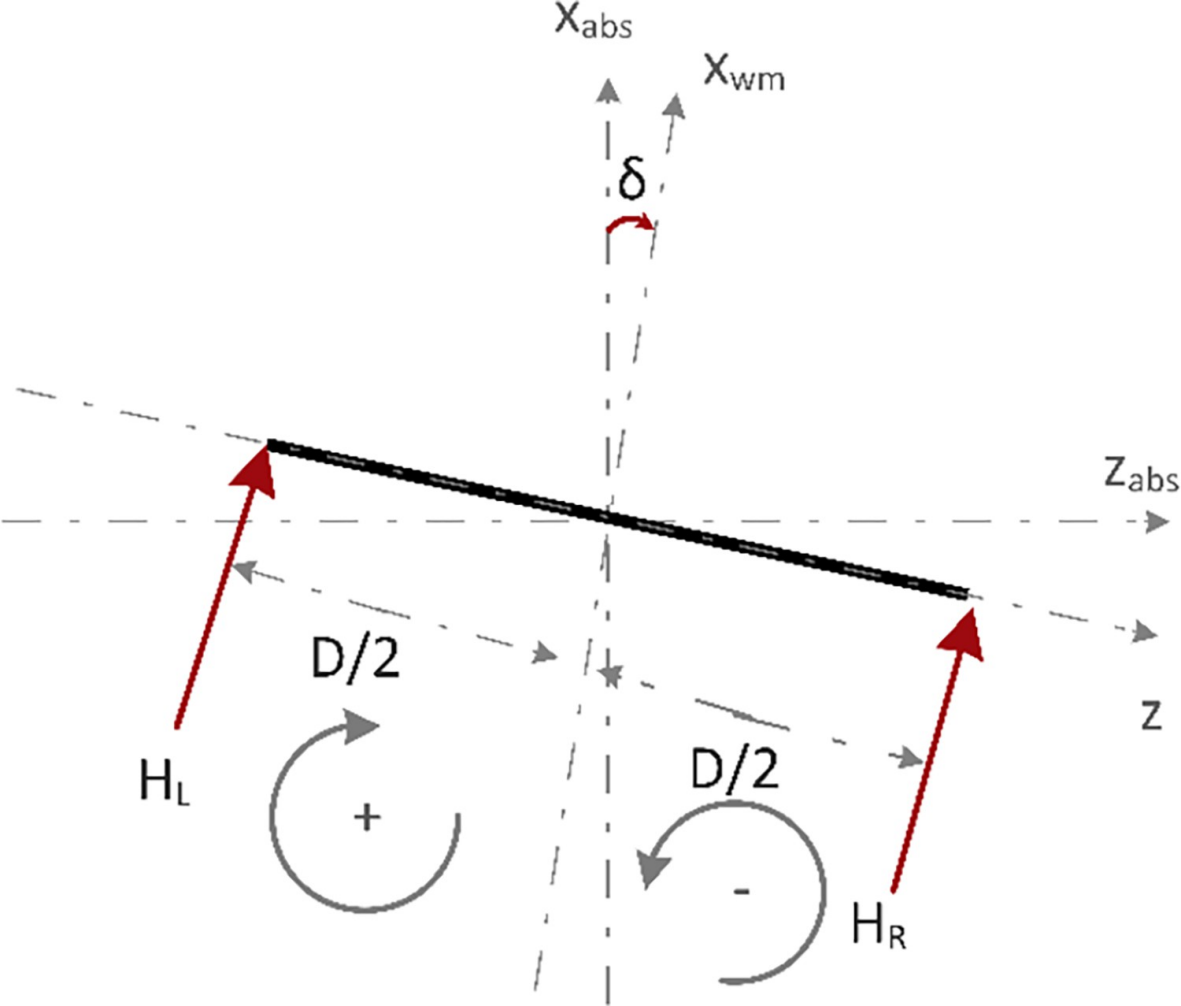
Torque diagram of the horizontal reaction forces between wheels and chassis about the y-axis.

Fig 4 shows the sum of torques acting on the chassis of the robot about the y-axis. With a moment arm of $\frac{D}{2}$, *H*_*L*_ and *H*_*R*_ generate clockwise and counterclockwise torques, respectively. Eq *(4)* gives the sum of torques acting on the chassis of the robot about the y-axis.

$$J_{\delta }\ddot{\delta }=H_{L}\frac{D}{2}-H_{R}\frac{D}{2}$$ (4)

OR

$$J_{\delta }\ddot{\delta }=(H_{L}-H_{R})\frac{D}{2}$$

Where

$$J_{\delta }=\frac{1}{3}M({\frac{D}{2})}^{2}=\frac{MD^{2}}{12}$$ (5)

All the forces are directed towards the right-hand side. Hence, using Newton’s second law of motion, sum of these forces can be represented by Eq *(6)*.


$$M\ddot{x}=f_{dp}+H_{L}+H_{R}$$ (6)

**Fig 5:**
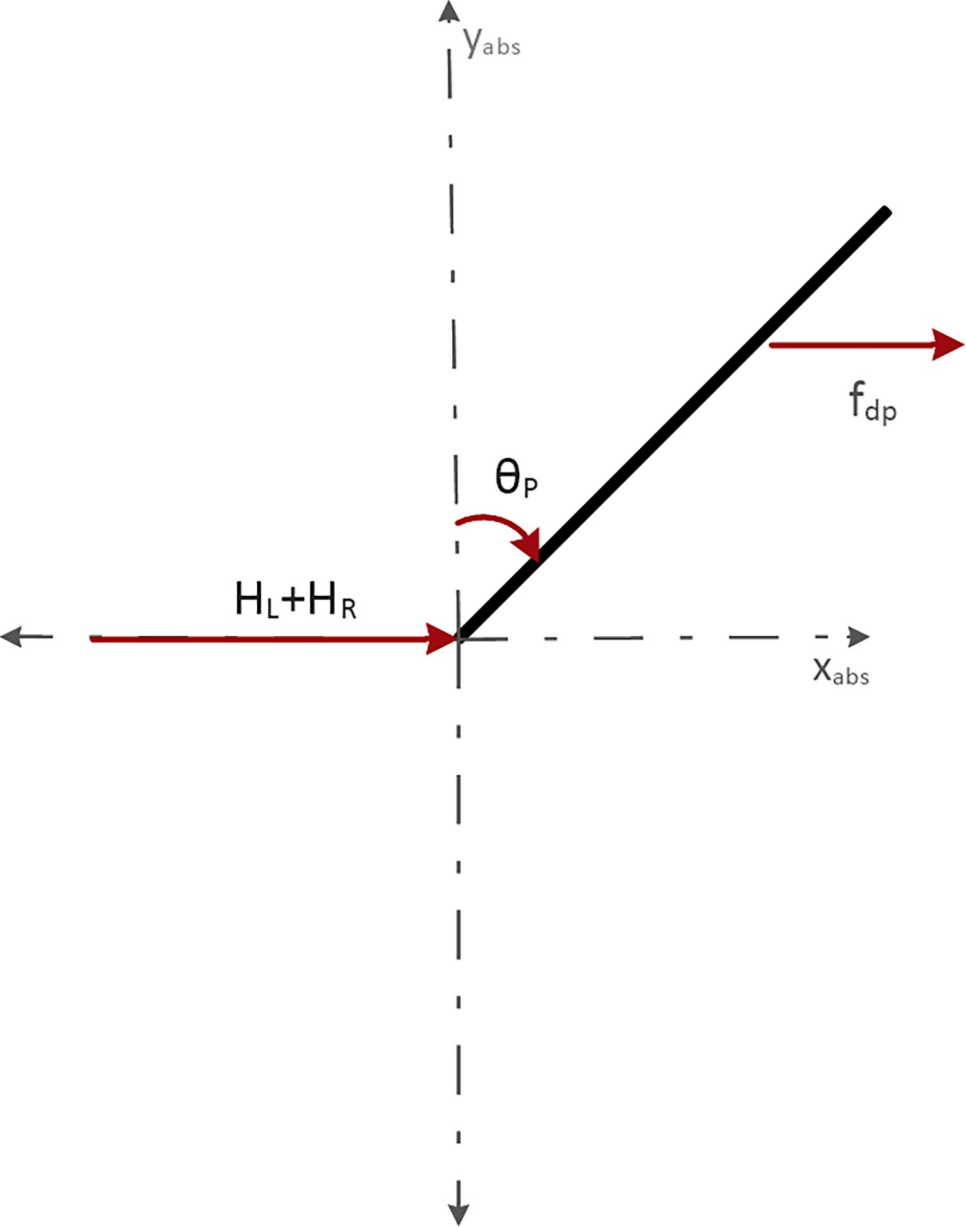
Force vector diagram of the horizontal reaction forces between wheels & chassis, and the disturbance force at the COG of the robot.

Fig *5* shows the force vector diagram of the horizontal reaction forces between wheels and chassis, and the disturbance force at the COG of the robot.

**Fig 6:**
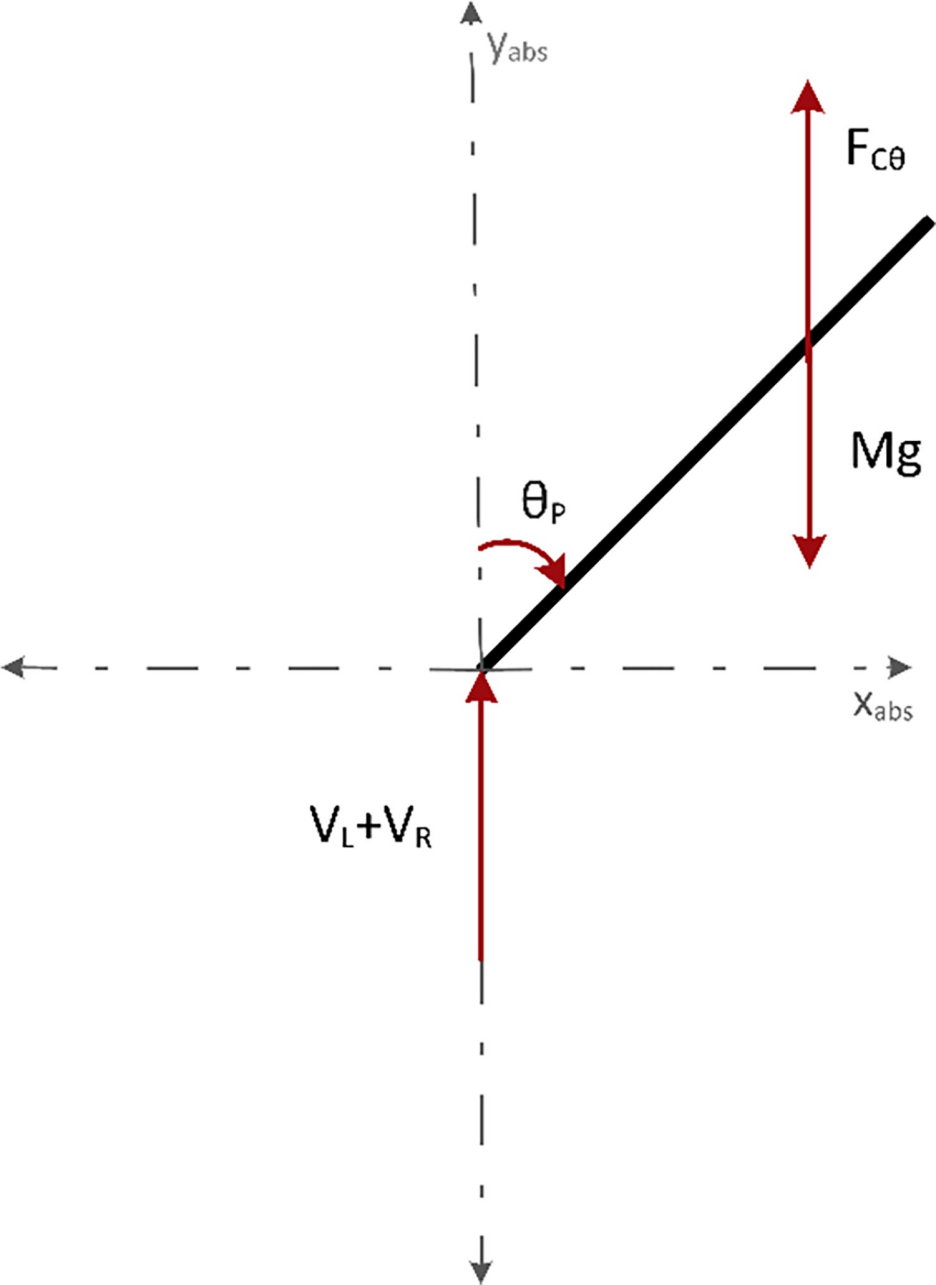
Force vector diagram of the vertical reaction forces between wheels and chassis, F_Cθ_, M_g_ acting at the COG.

Fig 6 shows the force vector diagram of the vertical reaction forces between wheels and chassis. *F*_*Cθ*_, *M*_*g*_ are acting at the COG. These forces act on the chassis along the y-axis. Upward forces were taken as positive, whereas downward forces as negative. Using this convention, the sum of forces on the chassis along the y-axis are given by Eq (7).


$$M\ddot{y}=V_{L}+V_{R}-M_{g}+F_{C\theta }$$ (7)

**Fig 7:**
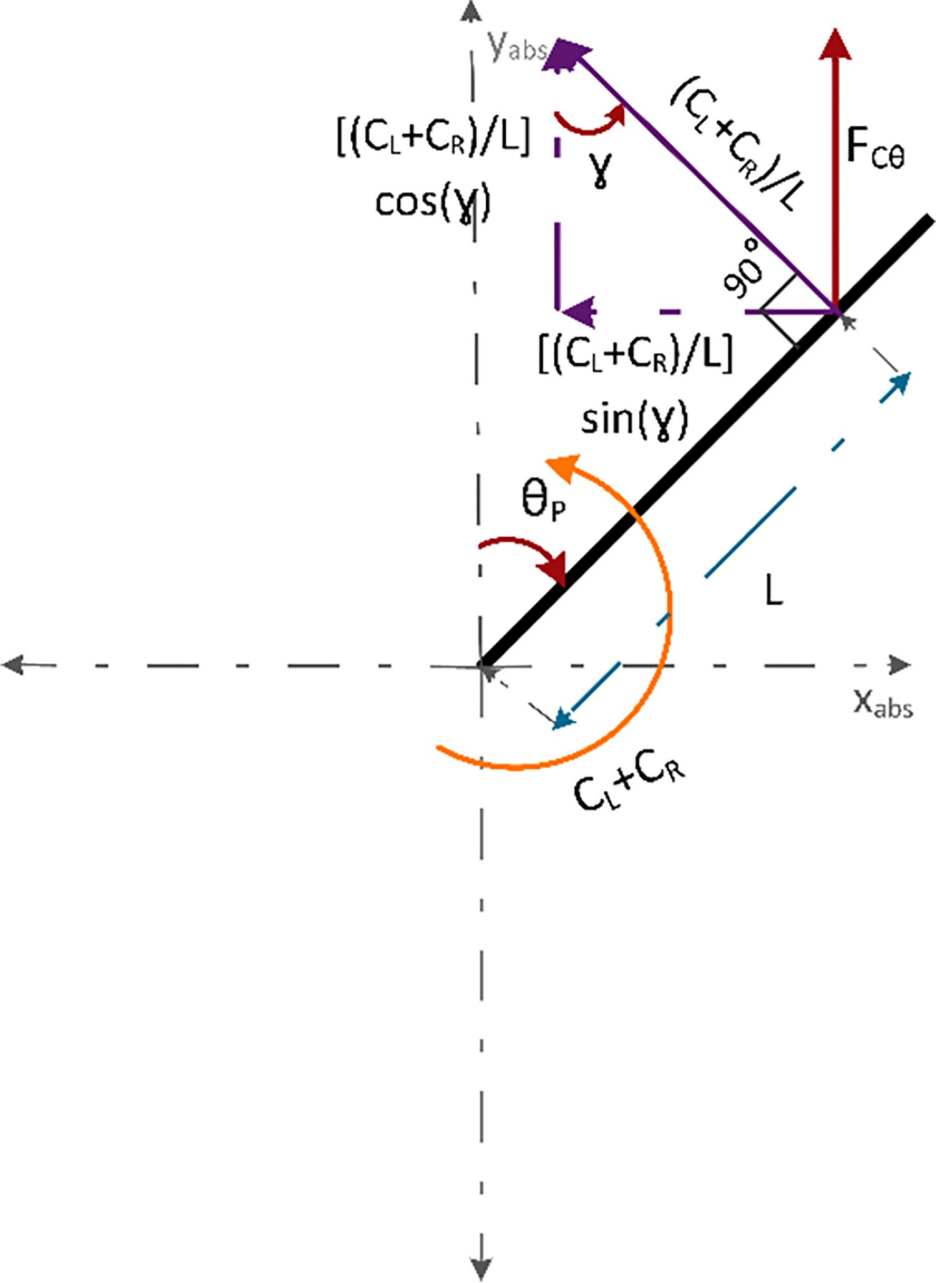
Force vector diagram of the components of F_Cθ_.

Fig 7 demonstrates the force vector diagram of the components of *F*_*Cθ*_. Ratio of sum of torque and moment arm results in the force acting at the COG i.e. $\frac{\left(C_{L}+C_{R}\right)}{L}$. The *cosine* component of this force is same as F_Cθ_. Where *γ* = 180−90−θ_P_ reduces to *γ* = 90−θ_P_. Since cos (90−*θ*_*P*_) = sin *θ*_*P*_, therefore, F_Cθ_ can be written by using Eq *(8)*.


$$F_{C\theta }=\frac{\left(C_{L}+C_{R}\right)}{L}sin(\theta_{P})$$ (8)

F_Cθ_ is substituted in Eq *(6)* to yield Eq *(9)*.


$$M\ddot{y}=V_{L}+V_{R}-M_{g}+\frac{\left(C_{L}+C_{R}\right)}{L}sin(\theta_{P})$$ (9)

**Fig 8:**
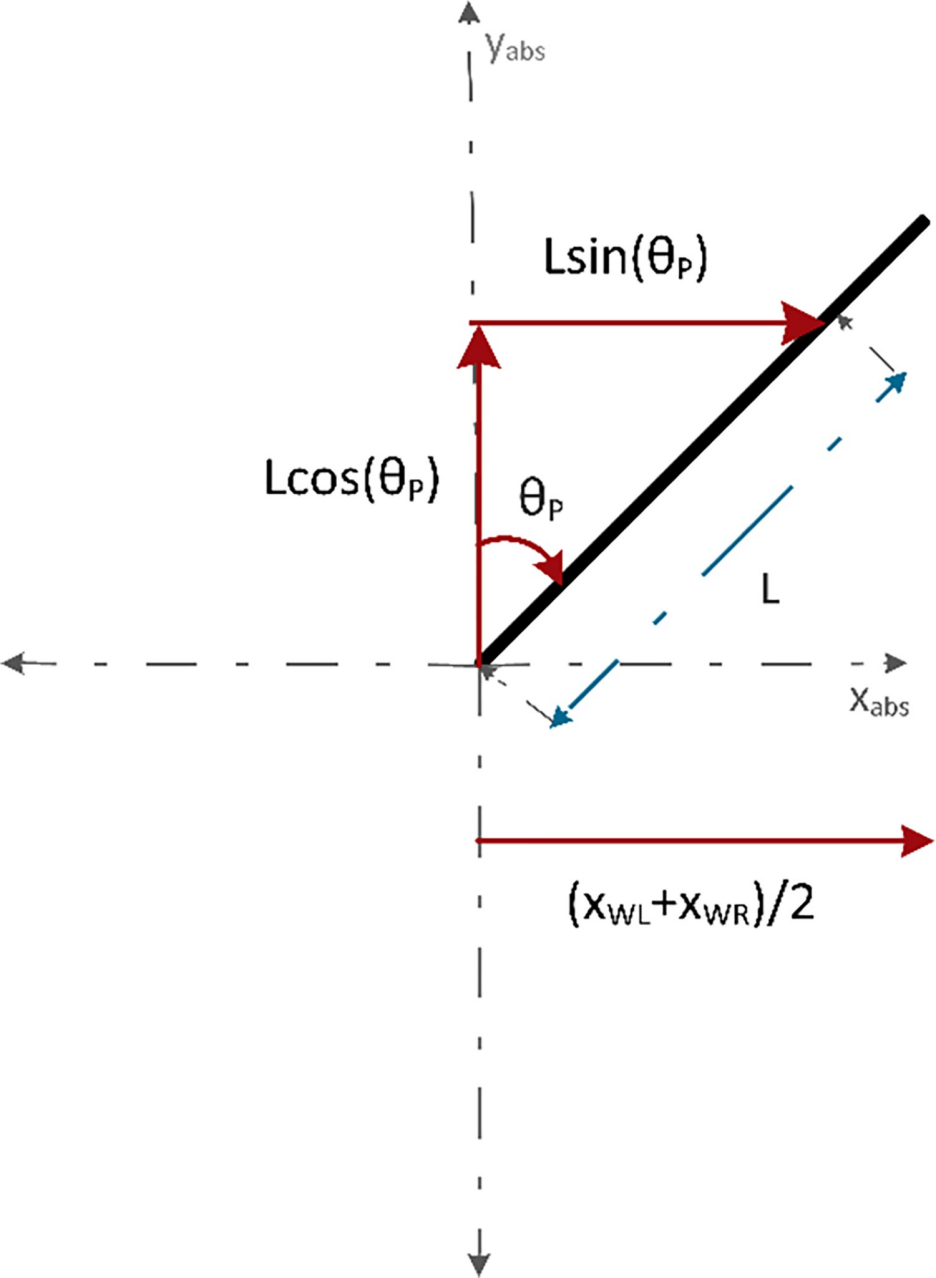
Force vector diagram representing the components of L for the distance traveled along the x-axis.

Fig 8 shows a force vector diagram representing the components of L for the distance traveled by the chassis along the x-axis. Eq *(10)* gives the distance traveled by the chassis that was equal to the sum of the average distance traveled by the wheel circumference and the *sin* component of the distance of COG from the z-axis.


$$x=L\;sin\;(\theta_{P})+\frac{x_{WL}+x_{WR}}{2}$$ (10)

**Fig 9:**
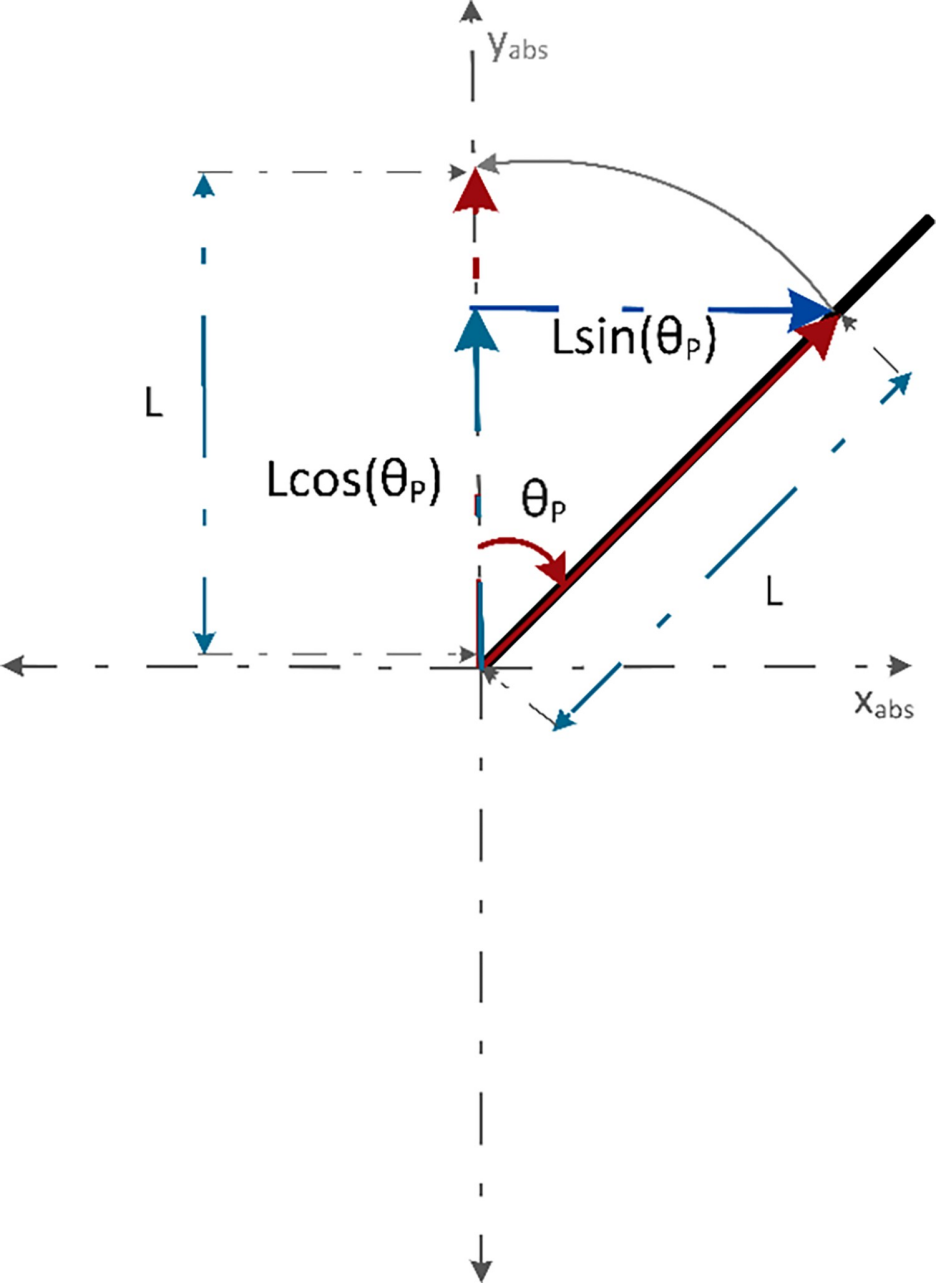
Force vector diagram representing the components of L for the distance traveled along the y-axis.

Fig 9 depicts the force vector diagram representing the components of L for the distance traveled along the y-axis. It has been assumed that the wheels of the robot do not leave the surface but the movement of the handlebar along the y-axis can be associated with its changing height due to the change in tilt angle. It could be seen that with the change in angle θ_P_, the component of length *Lcos*(θ_P_) that is along the y axis, changes as well. Hence, the displacement in the y-direction at any point can be given by the difference between the total length and the component of length along the y-axis.

Fig 2(A) and 2(B) represent the free body diagram of the left and the right wheel, respectively. These diagrams are used to represent the sum of torques and forces in Eqs *(11)*–*(20)*. Forces acting at moment arm generate torque along the axis of rotation of the wheels. Using Newton’s second law for rotational bodies, the sum of these moments for the left and right wheels are represented in Eqs *(11)* and *(12)*.

$$J_{wl}{\ddot{\theta }}_{WL}=C_{L}-H_{TL}R$$ (11)

and

$$J_{wr}{\ddot{\theta }}_{WR}=C_{R}-H_{TR}R$$ (12)

The generated torque rolls the wheel and covers a distance. It may be represented as the sector of the wheel having a radius *R*. The sector length for both wheels is given by Eqs *(14)* and *(15)*

$$y=-L-(-L\;\cos\;\theta_{P})$$ (13)

or

$$y=-L(1-\cos\;\theta_{P})$$

$$x_{WL}=\theta_{WL}R$$ (14)

$$x_{WR}=\theta_{WR}R$$ (15)

Using Newton’s second law of motion, sum of forces acting on the wheel along the x-axis are given in Eqs *(16)* and *(17)*.

$$M_{W}{\ddot{x}}_{WL}=f_{dl}+H_{TL}-H_{L}$$ (16)

and

$$M_{W}{\ddot{x}}_{WR}=f_{dR}+H_{TR}-H_{R}$$ (17)

Similarly, Eqs *(18)* and *(19)* represent the sum of forces along the y-axis.

$$M_{W}{\ddot{y}}_{WL}=V_{TL}-M_{W}g-V_{L}$$ (18)

and

$$M_{W}{\ddot{y}}_{WR}=V_{TR}-M_{W}g-V_{R}$$ (19)

Linear movement of chassis are characterized by the position *x*_*wm*_ that is the average of the sector length covered by the circumference of two wheels. This can be written as in Eq *(20)*.


$$x_{wm}=\frac{x_{WL}+x_{WR}}{2}$$ (20)

**Fig 10:**
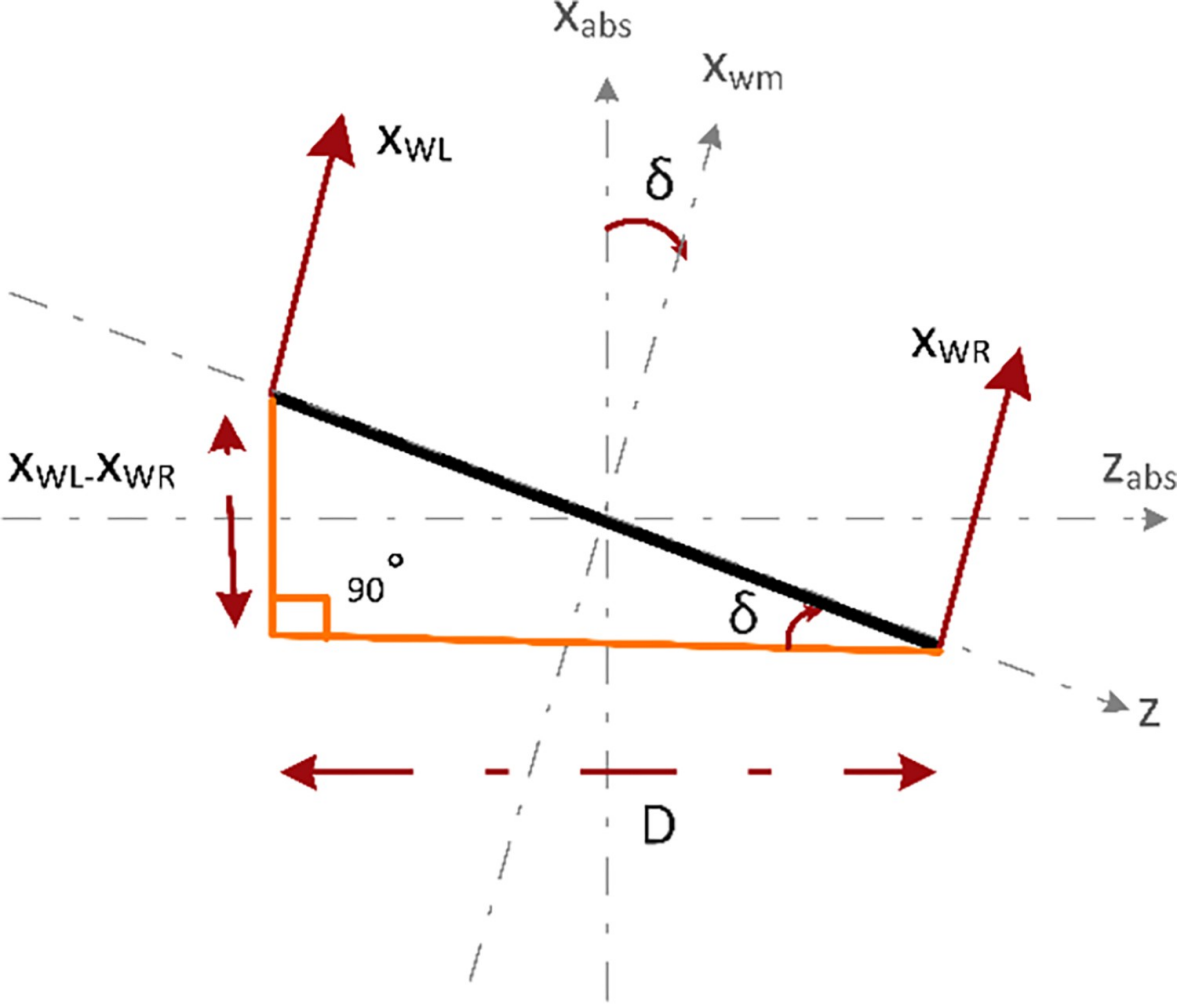
Force vector diagram of the chassis representing the yaw angle δ.

Fig 10 shows the force vector diagram of the chassis representing the yaw angle (*δ*). The robot has a yaw rotation when there is a difference in the rotation rate of the wheels. Yaw angle (*δ*) can be represented in terms of difference between the distance covered by the two wheels. Eq *(22)* is used to represent the yaw angle *δ*.

$$\tan\left(\delta \right)=\frac{x_{WL}-x_{WR}}{D}$$ (21)

or

$$\delta ={tan}^{-1}[\frac{x_{WL}-x_{WR}}{D}]$$ (22)

where *δ* is positive for X_WL_>X_WR_ and vice versa.

The wheels are characterized as circular discs and both have the same mass and radius. Therefore, their moment of inertia can be expressed by Eq *(23)*.


$$J_{w}=J_{wl}=J_{wr}=\frac{1}{2}M_{W}R^{2}$$ (23)

The total moment of inertia of the system is characterized as the sum of the rider’s moment of inertia and the chassis moment of inertia. The robot’s moment of inertia is calculated using an Autodesk inventor model of the robot and has turned out to be equal to 1.49 *kgm*^2^. Rider’s moment of inertia is approximated as that of a cylinder to simplify the model. As the moment of inertia for a cylindrical body can be represented as $\frac{1}{3}ML^{2}$, therefore, the total moment of inertia of the system is represented by Eq *(24)*.


$$J=J_{r}+J_{v}$$ (24)

Where

$$J_{v}=1.49$$ (25)

and

$$J_{r}=\frac{1}{3}M_{R}{L_{r}}^{2}$$ (26)

**Fig 11:**
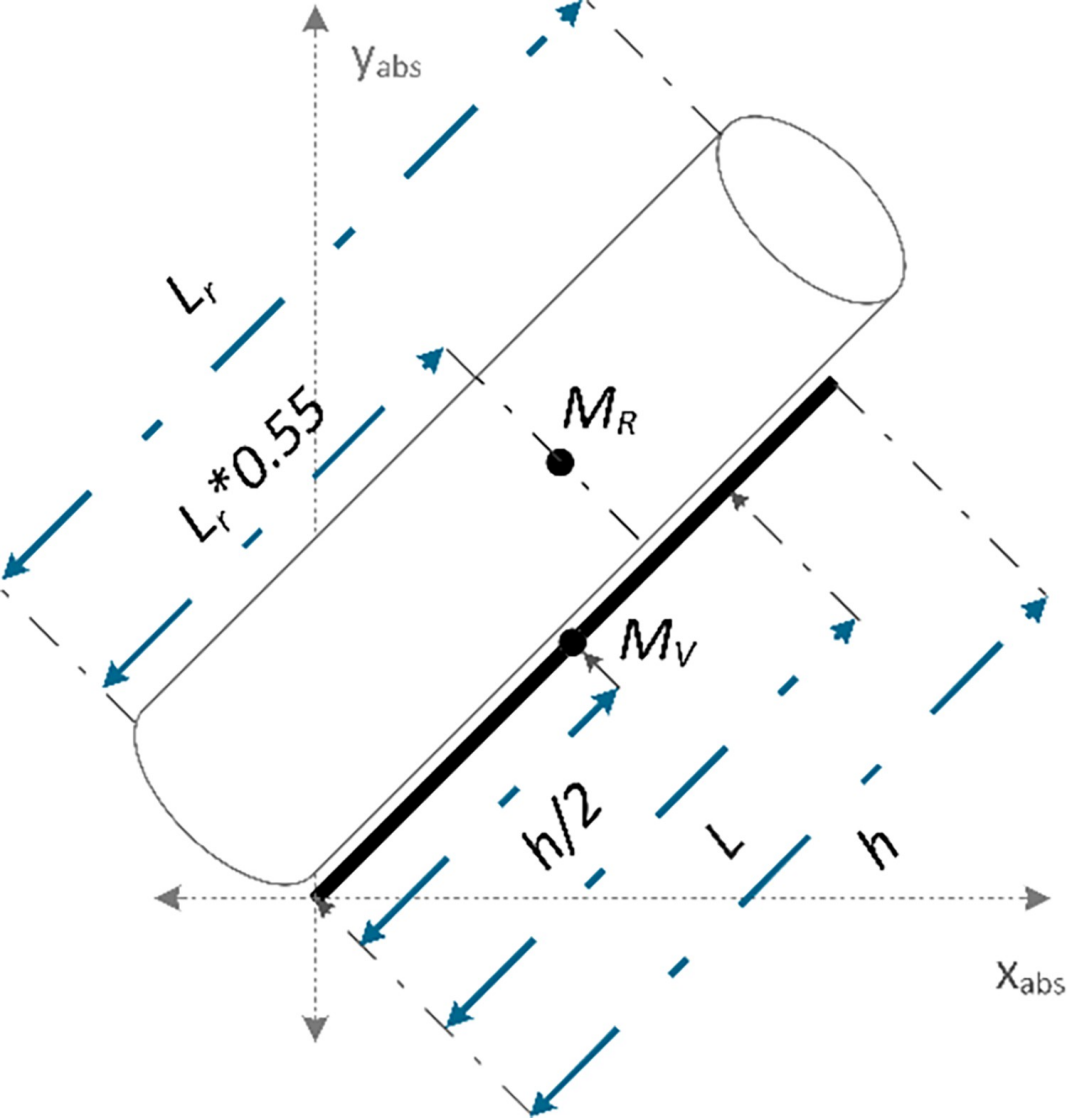
Free body diagram of the rider and its contribution to the value of L.

Fig 11 shows a free body diagram of the rider and its contribution to the value of L. This distance is represented using the center of mass equation with two-point masses. Mass of the rider *M*_*R*_ is represented as a point mass at 0.55 *L*_*r*_. This value is chosen because the center of gravity (COG) for an average human being lies around that height. Similarly, the mass of the robot *M*_*V*_ is represented as a point mass at the center of the rod $\frac{h}{2}$. The sum of these two-point masses was characterized as *M* = *M*_*R*_+*M*_*V*_ yielding the following Eq *(27)*.


$$L=\frac{\left(M_{V}\frac{h}{2}+M_{R}0.55L_{r}\right)}{M}$$ (27)

**Fig 12:**
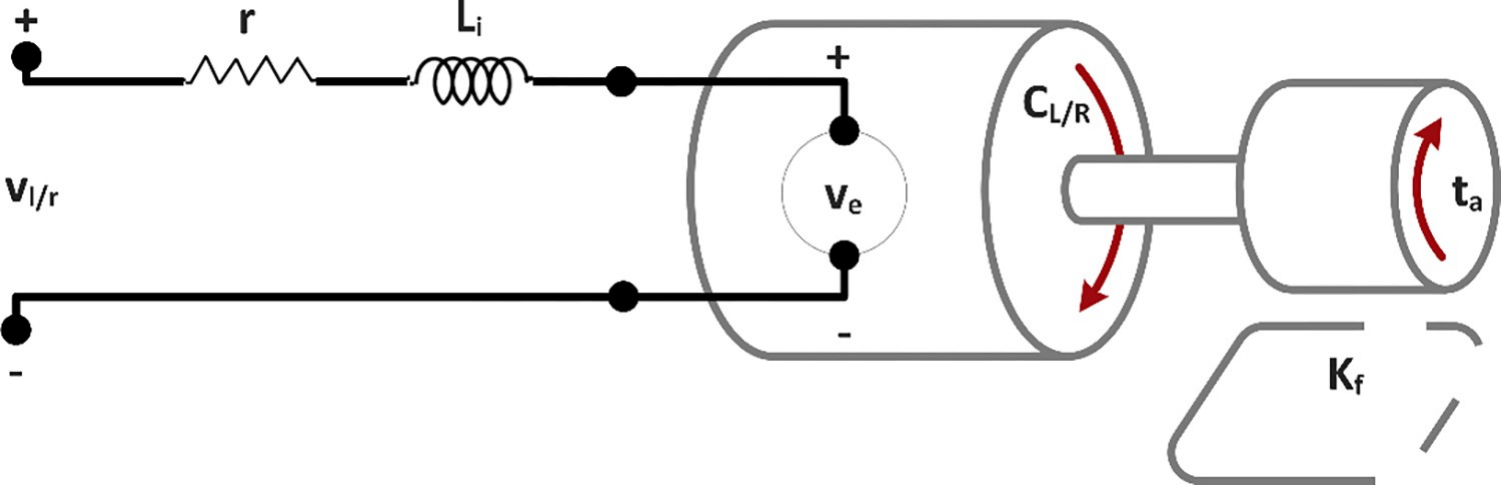
Schematic diagram of a direct current motor.

Fig 12 represents an effective model of a direct current motor. When voltage (*v*_*l*/*r*_) is applied to the motor terminals, current (*i*) flows in its armature. This current passes through the resistor (*r*) inductor (*L*) pair and generates a torque (*C*_*L*/*R*_). This torque is represented by Eq *(28)*.


$$C_{L/R}=k_{m}i$$ (28)

The current carrying coil of the motor is placed in a magnetic field and hence generates a back emf *v*_*e*_. This back emf can be approximated as a linear function of the velocity of the robot’s wheel.


$$v_{e}=K_{e}{\dot{\theta }}_{WL/WR}$$ (29)

Application of Kirchhoff’s voltage law on the model results in Eq *(30)*.


$$v_{l/r}-ri-L_{i}\frac{di}{dt}-v_{e}=0$$ (30)

Newton’s second law of motion states that the sum of all the torques acting on the shaft is linearly related to its acceleration by the inertial Load *J*_*R*_. The preceding statement is written in Eq *(31)*.


$$C_{L/R}-K_{f}{\dot{\theta }}_{WL/WR}-t_{a}=J_{R}{\dot{\theta }}_{WL/WR}$$ (31)

Where, *K*_*f*_ and *t*_*a*_ are taken as the frictional constant of the motor and load torque on the motor shaft, respectively.

To yield the differential equations that can represent the dynamics of the SBR, all the preceding equations are utilized. Eqs *(6)* and *(9)* were substituted into Eq *(3)* to yield Eq *(32)*.


$$J{\ddot{\theta }}_{P}=ML(\ddot{y}\;\sin\left(\theta_{P}\right)-\ddot{x}\;\cos\left(\theta_{P}\right))+MgL\;\sin\left(\theta_{P}\right)+f_{dp}\;\cos\left(\theta_{P}\right)-(C_{L}+C_{R})(1+\sin^{2}\left(\theta_{P}\right))$$ (32)

Eq *(20)* is substituted into Eq *(10)* and the double derivative of the resultant equation is calculated. After multiplying cos (*θ*_*P*_) on both sides of the equation, Eq *(33)* is deduced.


$$\ddot{x}\;\cos\left(\theta_{P}\right)=-L\;\sin\left(\theta_{P}\right)\;\cos\left(\theta_{P}\right){\dot{\theta_{P}}}^{2}+L\;\cos^{2}\left(\theta_{P}\right)\;{\ddot{\theta }}_{P}+{\ddot{x}}_{wm}\;cos(\theta_{P})$$ (33)

Eq *(34)* was derived by multiplying sin(*θ*_*P*_) on both sides of the double derivative of Eq *(13)*

$$\ddot{y}\;\sin\left(\theta_{P}\right)=-L\;\sin\left(\theta_{P}\right)\;\cos(\theta_{P})\;\dot{\theta_{P}}-L\sin^{2}\left(\theta_{P}\right){\ddot{\theta }}_{P}$$ (34)

Eq *(35)* can be obtained by solving for the difference of Eqs *(33)* and *(34)*

$$\ddot{y}\;\sin\left(\theta_{P}\right)-\ddot{x}\;\cos(\theta_{P})=-L{\ddot{\theta }}_{P}-{\ddot{x}}_{wm}cos\;(\theta_{P})$$ (35)

Eqs *(24)* and *(35)* are substituted into Eq *(34)*. The resultant equation can be represented by Eq *(36)*.


$$\left(\frac{1}{3}M_{R}{L_{r}}^{2}+1.49+ML^{2}){\ddot{\theta }}_{P}+ML{\ddot{x}}_{wm}\;\cos\;\left(\theta_{P}\right)=MgL\;\sin\left(\theta_{P}\right)+f_{dp}L\;\cos\left(\theta_{P}\right)-(C_{L}+C_{R})(1+\sin^{2}\left(\theta_{P}\right)\right)$$ (36)

Eq *(37)* is inferred from Eqs *(16)* and *(17)*

$$M_{W}{(\ddot{x}}_{WL}+{\ddot{x}}_{WR})=(H_{TL}+H_{TR})+(f_{dl}+f_{dr})-(H_{L}+H_{R})$$ (37)

Eqs *(38)* and *(39)* are deduced by substituting Eqs *(14)* and *(15)* in Eqs *(11)* and *(12)*, respectively.


$$H_{TL}=\frac{C_{L}R-J_{wl}{\ddot{x}}_{WL}}{R^{2}}$$ (38)

$$H_{TR}=\frac{C_{R}R-J_{wr}{\ddot{x}}_{WR}}{R^{2}}$$ (39)

Eq *(6)*, *(20)*, *(23)*, *(38)* and *(39)* are substituted into Eq *(37)*. The resultant equation can be represented as follows.


$$2M_{W}{\ddot{x}}_{wm}=-M\ddot{x}+f_{dp}+\frac{{(C}_{L}+C_{R})}{R}-\frac{2J_{w}{\ddot{x}}_{wm}}{R^{2}}+f_{dl}+f_{dr}$$ (40)

Eq *(20)* is substituted into Eq *(10)* and double derivative of the resultant equation is evaluated. The resultant equation is formulated as

$$\ddot{x}=L\;\cos\left(\theta_{P}\right){\ddot{\theta }}_{P}-L\;\sin\left(\theta_{P}\right){\dot{\theta_{P}}}^{2}+{\ddot{x}}_{wm}$$ (41)

Eq *(41)* is substituted in Eq *(40)*. The resultant equation yields as Eq *(42)*.


$$3(M_{W}+M{)\ddot{x}}_{wm}=ML\;\sin(\theta_{P})\;{\dot{\theta_{P}}}^{2}-ML\;\cos\left(\theta_{P}\right)\;{\ddot{\theta }}_{P}+\frac{{(C}_{L}+C_{R})}{R}$$ (42)

For an effective model including motor dynamics, a relationship between motor voltages *v*_*l*_, *v*_*r*_ and the control torque of the wheel *C*_*L*_, *C*_*R*_ is derived. Eq *(29)* is substituted into Eq *(30)*. The resultant equation is rearranged and written as Eq *(43)*.


$$i=\frac{v_{l/r}}{r}-\frac{K_{e}{\dot{\theta }}_{WL/WR}}{r}$$ (43)

Here, inductance *L*_*i*_ of the motor is assumed to be zero. Eq *(28)* is substituted into Eq *(31)* to yield Eq *(44)*.


$$C_{L/R}=k_{m}i=J_{R}{\dot{\theta }}_{WL/WR}$$ (44)

Motor frictional constant *K*_*f*_ and applied torque on the motor *t*_*a*_ are approximated as zero. Eq *(43)* is substituted into Eq *(44)* resulting in Eq *(45)*

$$J_{R}{\dot{\theta }}_{WL/WR}=\frac{{k_{m}v}_{l/r}}{r}-\frac{{k_{m}K}_{e}{\dot{\theta }}_{WL/WR}}{r}$$ (45)

A combination of Eqs *(44)*, *(45)*, *(14)* and *(15)* is used to formulate following equations for the left and right motor of the SBR.

$$C_{L}=\frac{{k_{m}v}_{l}}{r}-\frac{{k_{m}K}_{e}{\dot{x}}_{WL}}{rR}$$ (46)

and

$$C_{R}=\frac{{k_{m}v}_{r}}{r}-\frac{{k_{m}K}_{e}{\dot{x}}_{WR}}{rR}$$ (47)

Equations for the pitch and yaw angle control torques *C*_*θ*_ and *C*_*δ*_ are formulated as functions of pitch and yaw angle control voltages *v*_*θ*_ and *v*_*δ*_, respectively. This has been done using Eqs *(46)*, *(47)*, *(20)* and *(22)*.

$$C_{\theta }=C_{L}+C_{R}=\frac{k_{m}}{r}(v_{\theta })-\frac{2{k_{m}K}_{e}}{Rr}({\dot{x}}_{wm})$$ (48)

and

$$C_{\delta }=C_{L}-C_{R}=\frac{k_{m}}{r}(v_{\delta })-\frac{{k_{m}K}_{e}}{Rr}(D\;\sec^{2}\left(\delta \right)\dot{\delta })$$ (49)

Where, *v*_*θ*_ = *v*_*l*_+*v*_*r*_ and *v*_*δ*_ = *v*_*l*_+*v*_*r*_.

Decoupling of these control terms lead to individual voltages for the left and the right wheel. This is done using the Eqs *(50)* and *(51)*.

$$v_{l}=\frac{v_{\theta }+v_{\delta }}{2}$$ (50)

and

$$v_{r}=\frac{v_{\theta }-v_{\delta }}{2}$$ (51)

System of Eqs *(33)* and *(42)* are solved simultaneously to formulate differential equations that represent dynamics of the system. Eq *(48)* is then substituted into the resultant differential equations to deduce Eqs *(52)* and *(53)*.

$${\ddot{\theta }}_{P}=\frac{C_{1}}{A}+\frac{B_{1}M_{2}}{A}({\dot{x}}_{wm})-\frac{B_{1}M_{1}}{A}(v_{\theta })-\frac{D_{\theta }}{A}$$ (52)

and

$${\ddot{x}}_{wm}=\frac{C_{2}}{A}-\frac{B_{2}M_{2}}{A}({\dot{x}}_{wm})+\frac{B_{2}M_{1}}{A}(v_{\theta })+\frac{D_{x}}{A}$$ (53)

Where

$$A=1.49M+4.47M_{W}+M^{2}L^{2}\;\sin^{2}\left(\theta_{P}\right)+\frac{M{L_{r}}^{2}M_{R}}{3}+3ML^{2}M_{W}+M_{W}{L_{r}}^{2}M_{R}$$

$$B_{1}=(M+3M_{W})(1+\sin^{2}\left(\theta_{P}\right))+\frac{ML\;\cos\left(\theta_{P}\right)}{R}$$

$$C_{1}=ML\;\sin\left(\theta_{P}\right)[(M+3M_{W})g-ML\;\cos\left(\theta_{P}\right){{\dot{\theta }}_{P}}^{2}]$$

$$B_{2}=\frac{1.49+ML^{2}+\frac{{L_{r}}^{2}M_{R}}{3}}{R}+ML\;\cos\left(\theta_{P}\right)(1+\sin^{2}\left(\theta_{P}\right))$$

$$C_{2}=ML\;\sin\left(\theta_{P}\right)[(1.49+ML^{2}+\frac{{L_{r}}^{2}M_{R}}{3}){{\dot{\theta }}_{P}}^{2}-MLg\;\cos\left(\theta_{P}\right)]$$

$$M_{1}=\frac{k_{m}}{r}$$

$$M_{2}=\frac{2{k_{m}K}_{e}}{Rr}$$

$$D_{\theta }=ML\;\cos\left(\theta_{P}\right)(f_{dl}+f_{dr})-3LM_{W}f_{dp}\;\cos\left(\theta_{P}\right)$$

$$D_{x}=f_{dp}(1.49+ML^{2}\;\sin^{2}\left(\theta_{P}\right)+\frac{{L_{r}}^{2}M_{R}}{3})+f_{dl}(1.49+ML^{2}+\frac{{L_{r}}^{2}M_{R}}{3})+f_{dr}(1.49+ML^{2}+\frac{{L_{r}}^{2}M_{R}}{3})$$

On the other hand, Eqs *(11)*, *(12)*,*(14)*,*(15)*,*(16)* and *(17)* are used to infer yaw angular acceleration.

$$H_{L}=\frac{C_{L}}{R}-{\ddot{x}}_{WL}(\frac{J_{wl}}{R^{2}}+M_{W})+f_{dl}$$ (54)

and

$$H_{R}=\frac{C_{R}}{R}-{\ddot{x}}_{WR}(\frac{J_{wr}}{R^{2}}+M_{W})+f_{dr}$$ (55)

Eq *(55)* is subtracted from Eq *(54)* to yield Eq *(56)*.

$$H_{L}-H_{R}=\frac{C_{\delta }}{R}-({\ddot{x}}_{WL-}{\ddot{x}}_{WR})(\frac{J_{w}}{R^{2}}+M_{W})+f_{dl}-f_{dr}$$ (56)

Eq *(57)* can be inferred from Eq *(22)*

$${\ddot{x}}_{WL-}{\ddot{x}}_{WR}=2\sec^{2}\left(\delta \right)\tan\left(\delta \right){\dot{\delta }}^{2}D+\sec^{2}\left(\delta \right)D\ddot{\delta }$$ (57)

Eqs *(5)*, *(23)*, *(49)* and *(57)* are substituted into Eq *(56)*. Eq *(58)* represents SBR’s dynamics about the y-axis.


$$\ddot{\delta }=\frac{12M_{1}}{A_{3}}v_{\delta }-\frac{12M_{3}}{A_{3}}\dot{\delta }-\frac{B_{3}}{A_{3}}+\frac{D_{\delta }}{A_{3}}$$ (58)

Where

$$A_{3}=2RDM+18RDM_{W}\sec^{2}\left(\delta \right)$$

$$B_{3}=36RDM_{W}\sec^{2}\left(\delta \right)\tan\left(\delta \right){\dot{\delta }}^{2}$$

$$M_{3}=\frac{K_{m}K_{e}}{Rr}D\;\sec^{2}\left(\delta \right)$$

$$D_{\delta }=12R(f_{dl}-f_{dr})$$

### Design of sliding mode controller for self-balancing robot

A nonlinear sliding mode control system is designed to regulate the pitch and yaw angle of the SBR. In practical systems, it may not be possible to measure the parameters of the system with reasonable accuracy. Which causes modeling error and as a result, the stability of the system is compromised. Therefore, it is important to ensure the stability of the system against modeling errors. To achieve this, EKF is used in coordination with the SMC, which increases the robustness of the system against modeling errors. SMC is a variable structure control method that alters the dynamics of the nonlinear system by applying a discontinuous control signal. This signal forces the system to "slide" along the system’s normal behavior. SMC design can be broadly categorized into two parts. The first part deals with the designing of a sliding surface with desirable attributes like tracking and stability. The second part entails the designing of discontinuous control law that makes the sliding surface an invariant set. This control law also ensures that the sliding surface has finite time reachability.

Sliding surface design requires the knowledge of estimation error. The estimation errors for pitch and yaw angle are defined in Eqs *(59)* and *(60)* as the difference between the measured and desired pitch & yaw angles, respectively.


$$\theta_{Pe}=\theta_{P}-\theta_{Pd}$$ (59)

$$\delta_{e}=\delta -\delta_{d}$$ (60)

Hence, the sliding surface is defined as the error based linear hyperplane shown in Eqs *(61)* and *(62)*

$$S_{\theta }={\dot{\theta }}_{Pe}+a_{\theta }\theta_{Pe}$$ (61)

$$S_{\delta }={\dot{\delta }}_{e}+a_{\delta }\delta_{e}$$ (62)

Where *a*_*θ*_ and *a*_*δ*_ are tuning variables that must be positive integers to ensure convergence of error to zero. After designing the sliding surfaces, an appropriate reaching law is designed. This law ensures the convergence of the measured angle to the sliding surface and keeps it there. Although SMC is a very efficient controller, using the sine function generally causes the problem of chattering. As a solution, a reaching law with *tanh* as shown in Eqs *(63)* and *(64)* is used.


$${\dot{S}}_{\theta }=-K_{\theta }\;\tanh\left(S_{\theta }\right)$$ (63)

$${\dot{S}}_{\delta }=-K_{\delta }\;\tanh\left(S_{\delta }\right)$$ (64)

Where *K*_*θ*_ and *K*_*δ*_ are controller gains for pitch and angle control, respectively. These gains can be tuned to achieve the desired performance. For the angles to reach the sliding surface, the reachability conditions must be satisfied i.e. $S_{\theta }{\dot{S}}_{\theta }<0$ and $S_{\delta }{\dot{S}}_{\delta }<0$. To validate these conditions, we have assumed ${\dot{S}}_{\theta }=-K_{\theta }\;\tanh\left(S_{\theta }\right)$ and ${\dot{S}}_{\delta }=-K_{\delta }\;\tanh\left(S_{\delta }\right)$. Controller equations for both pitch and yaw may be derived using the reaching law, sliding surface, and the estimation error. Substituting Eqs *(61)* and *(62)* into Eqs *(63)* and *(64)*, respectively, lead to Eqs *(65)* and *(66)*.


$${\ddot{\theta }}_{Pe}+a_{\theta }{\dot{\theta }}_{Pe}=-K_{\theta }\;\tanh\left(S_{\theta }\right)$$ (65)

$${\ddot{\delta }}_{e}+a_{\delta }{\dot{\delta }}_{e}=-K_{\delta }\;\tanh\left(S_{\delta }\right)$$ (66)

Eqs *(67)* and *(68)* are derived by substituting the double derivative of Eqs *(59)* and *(60)* into Eqs *(65)* and *(66)*, respectively.


$${\ddot{\theta }}_{P}-{\ddot{\theta }}_{Pd}+a_{\theta }{\dot{\theta }}_{Pe}=-K_{\theta }\;\tanh\left(S_{\theta }\right)$$ (67)

$$\ddot{\delta }-{\ddot{\delta }}_{d}+a_{\delta }{\dot{\delta }}_{e}=-K_{\delta }\;\tanh\left(S_{\delta }\right)$$ (68)

Substituting the values of ${\ddot{\theta }}_{P}$ and $\ddot{\delta }$ from Eqs *(52)* and *(58)* in Eqs *(67)* and *(68)*, respectively, then rearranging the controller equation for pitch and yaw angle, their respective control laws can be deduced. They are represented in Eqs *(69)* and *(70)*.


$$v_{\theta }=\frac{{K_{\theta }\;\tanh\left(S_{\theta }\right)+D_{1}-{\ddot{\theta }}_{Pd}+a}_{\theta }{\dot{\theta }}_{Pe}}{E_{1}}$$ (69)

Where

$D_{1}=\frac{C_{1}}{A}+\frac{B_{1}M_{2}}{A}({\dot{x}}_{wm}),\;E_{1}=\frac{B_{1}M_{1}}{A}$ and it is assumed that *D*_*θ*_ = 0

$$v_{\delta }=\frac{-{K_{\delta }\;\tanh\left(S_{\delta }\right)-D_{3}+{\ddot{\delta }}_{d}-a}_{\delta }{\dot{\delta }}_{e}}{E_{3}}$$ (70)

Where

$D_{3}=-\frac{12M_{3}}{A_{3}}\dot{\delta }-\frac{B_{3}}{A_{3}},\;E_{3}=\frac{12M_{1}}{A_{3}}$ and it is assumed that *D*_*δ*_ = 0.

EKF algorithm is a nonlinear extension of the Kalman filter. It gets the first-order linear approximation of the nonlinear system at each sampling time. For this sampling time, it acts as a linear Kalman filter. It is utilized widely in several real-world applications for the estimation of unknown and un-measurable system states, unknown parameters, faults, and disturbances.

**Fig 13:**
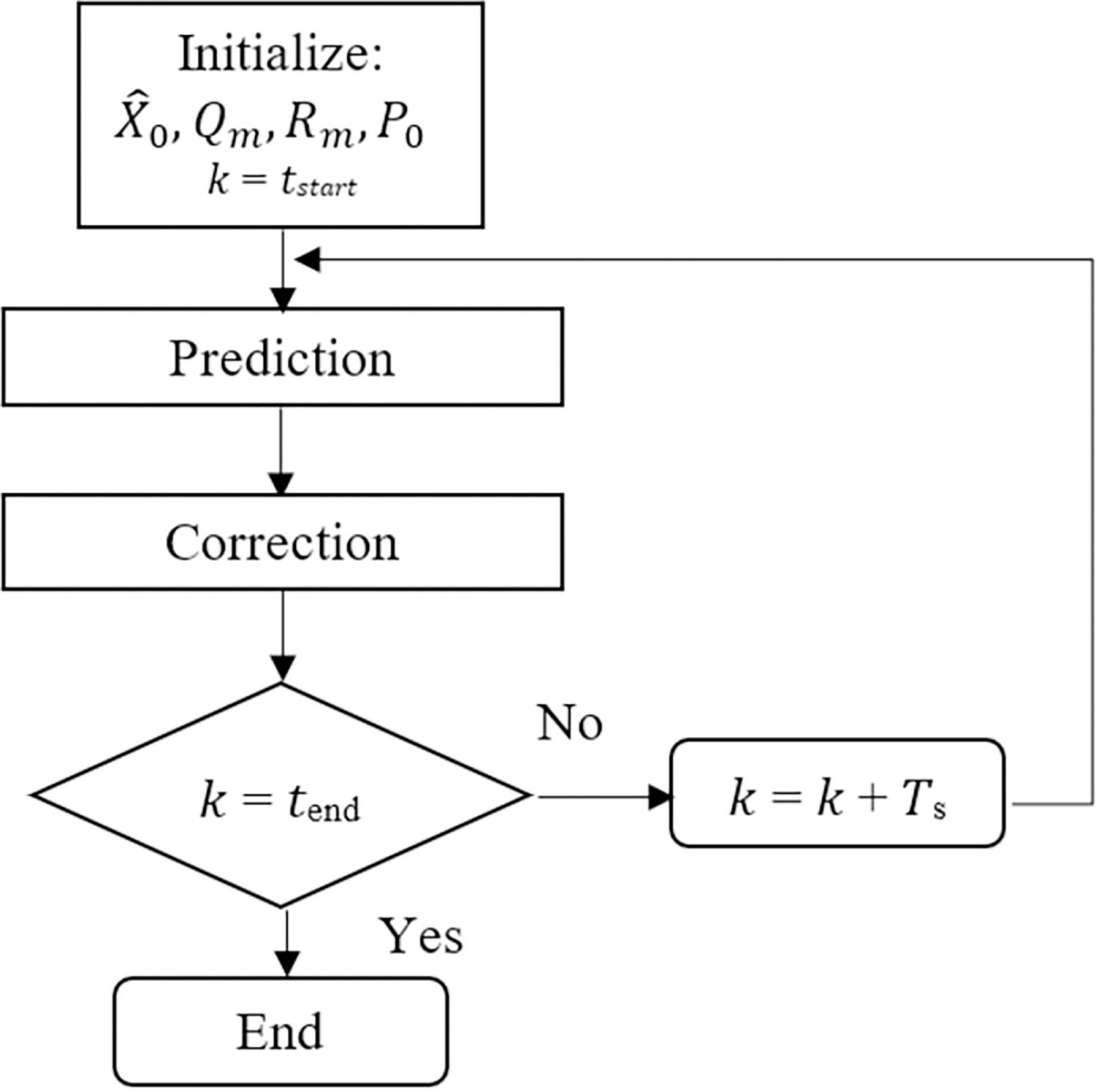
Flowchart for the extended Kalman filter algorithm.

In this proposed technique, riders’ mass *M*_*R*_ and length *L*_*r*_ are estimated. This estimation is based on the convergence of the measured and calculated state values. EKF algorithm implementation is divided into two steps: prediction and correction. The process noise and measurement noise covariance matrixes are represented by *Q*_*m*_ and *R*_*m*_, respectively. *P*_*k*_ is the state error covariance matrix. Fig 13 shows the flowchart for prediction and correction steps for each sampling instant. Where

$$\hat{X}=[\theta_{P},{\dot{\theta }}_{P},x_{wm},{\dot{x}}_{wm},\delta ,\dot{\delta },{\hat{M}}_{R},{\hat{L}}_{r}].$$

$\hat{X}$ represents a modified SBR model state vector consisting of 8 states. The stopping criteria of the algorithm depend on the run time and sampling rate of the simulation. Stopping time (t_*end*_) can be defined by Eq *(71)*

$$t_{end}=t_{start}+\frac{Run\;time}{T_{s}}$$ (71)

Prediction is carried out in two steps. In the first step, states are predicted for the next sampling instant using the previous state estimates. In the second step, the state error covariance matrix is predicted for the next sampling instant using the model Jacobian matrix and process noise covariance matrix. Correction is accomplished in three steps. In the first step, Kalman filter gain K is calculated using the predicted state error covariance matrix. In the second step, states are corrected using available measurements and Kalman gain. In the third step, the prediction of the state error covariance matrix is made.

### Proposed algorithm for SBR control system design

**Fig 14:**
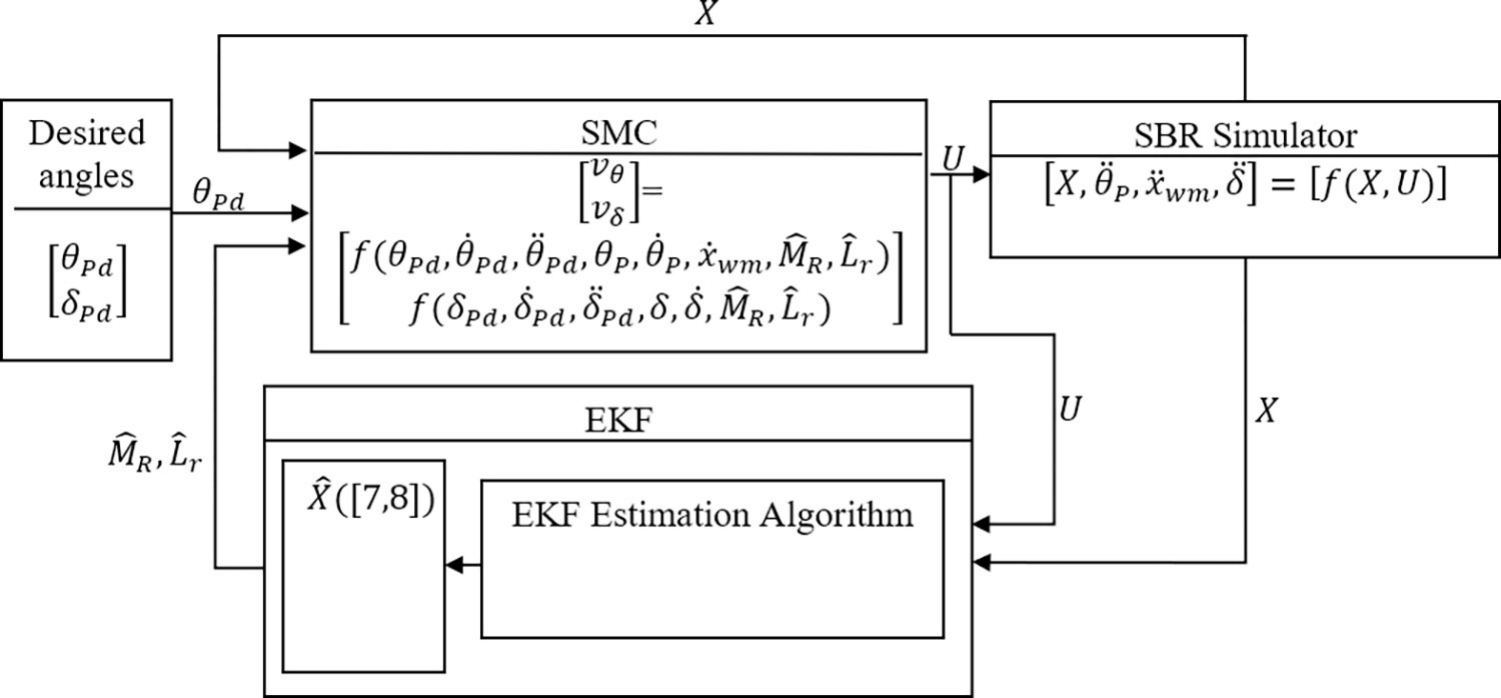
Block diagram representation of the proposed algorithm for EKF based SMC.

Modeling mismatch affects the performance of the control system; hence, estimation of model uncertainties ensures the improved performance of SMC beyond the given bounds in the literature. An Extended Kalman Filter is used to estimate model uncertainties of ${\hat{M}}_{R}$ and ${\hat{L}}_{r}$. Fig 14 shows the block diagram for the proposed Extended Kalman Filter based Sliding Mode Controller (ESMC). The desired pitch ***θ***_***Pd***_ and yaw angle ***δ***_***d***_ are fed to the SMC. The value of ***θ***_***Pd***_ is kept zero and ***δ***_***d***_ changes according to the turn command given by the rider. For the purpose of simulation, it is taken as a sinusoidal signal. The states calculated by the differential equations in the SBR simulator are also fed to the SMC. These states are represented as $X=[\theta_{P},{\dot{\theta }}_{P},x_{wm},{\dot{x}}_{wm},\delta ,\dot{\delta }]$. EKF estimates the values of ${\hat{M}}_{R}$ and ${\hat{L}}_{r}$. These values go into SMC as inputs as well. SMC utilizes these inputs to generate the control effort ***U*** = [***v***_***θ***_, ***v***_***δ***_]. This is provided to the SBR simulator containing the dynamic equations of the system, ensuring its stability as per the desired angles. EKF takes ***X*** and ***U*** as inputs and estimates the values of rider’s mass ${\hat{M}}_{R}$ and length ${\hat{L}}_{r}$.

**Fig 15:**
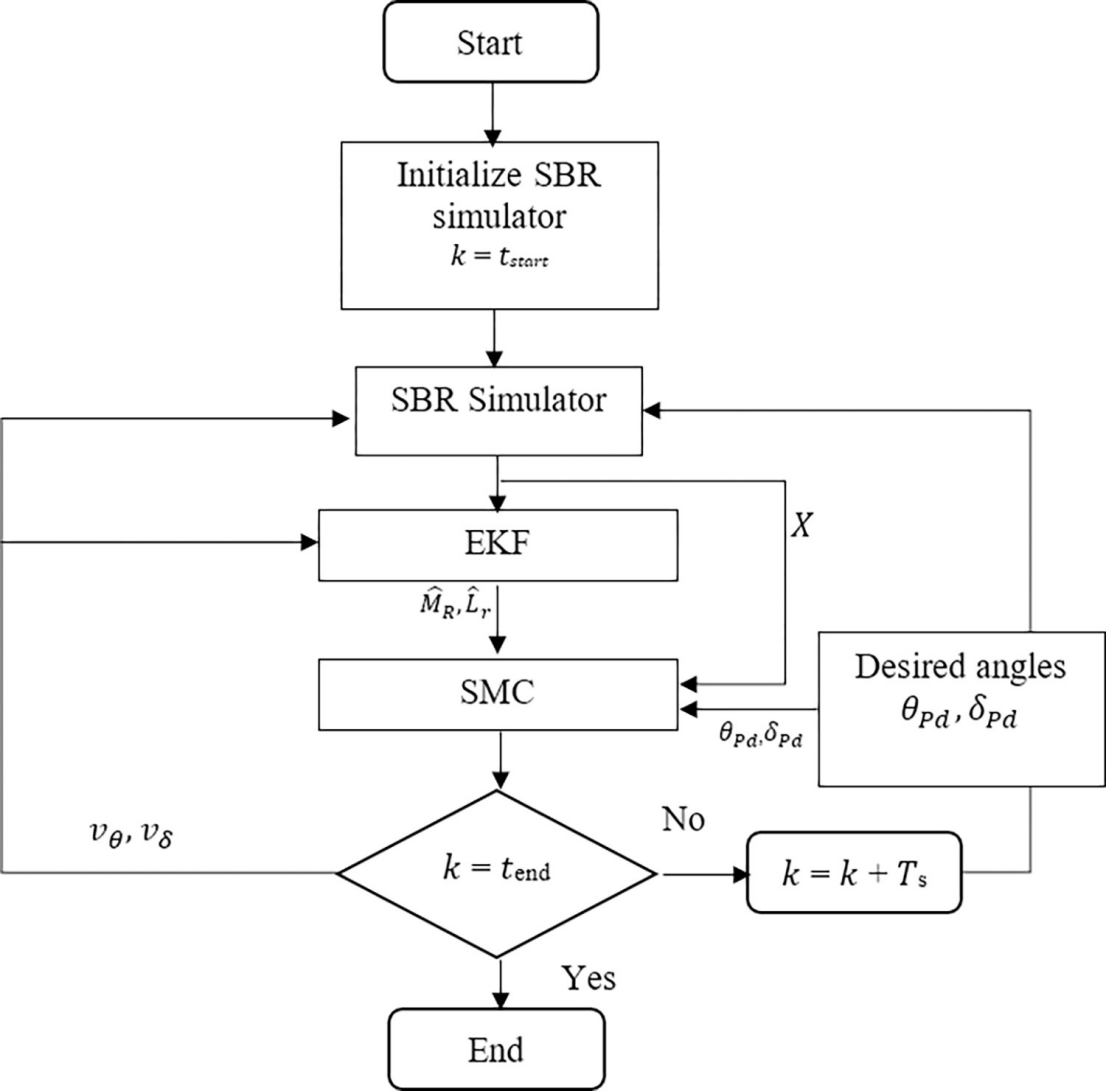
Flow chart for the proposed SBR’s stability control algorithm.

Fig 15 gives the flow chart of the proposed algorithm for efficient regulation of desired angles and stability. The SBR simulator is initialized with initial states ***X***_**0**_. In the next step SBR simulator receives the control inputs ***U***_**0**_. As the system under examination is an underactuated one, it tends to destabilize. Resulting states of the system ***X*** along with the inputs ***U***_**0**_ are fed to the EKF. The dynamic model of the SBR implemented in EKF contains the nominal values of the rider’s mass and length. EKF calculates the system’s states $\hat{X}([1:6])$ based on these nominal values. It then compares them to the states ***X*** measured by the SBR simulator which contains the actual information of the rider’s mass and length in its dynamic model equations. Since both EKF and the SBR simulator use the same inputs at any given time, the difference between $\hat{X}(\left[1:6\right])$ and ***X*** states can only be justified by some unknown value. In this proposed method, all disturbances are ignored due to its limitations for the indoor environment applications. However, variations due to the rider’s mass ***M***_***R***_ and length ***L***_***r***_ are examined. EKF tries to minimize the difference between the calculated states by estimating these values. The SMC block implements the proposed stability control method.

It utilizes the estimates of EKF, calculated system states ***X***, and the desired angles and their derivative to calculate the control action necessary to attain system stability. It is then fed back to the EKF and SBR simulator to start this process all over again. This loop runs until the criteria given in Eq *(71)* is satisfied. With each iteration, EKF tends to converge its estimate (${\hat{M}}_{R}$ and ${\hat{L}}_{r}$) to the actual values of ***M***_***R***_ and ***L***_***r***_.

Two types of tuning parameters are used in this manuscript. The first refers to the controller gains that include the sliding surface gains (*a*_*θ*_, *a*_*δ*_) and the switching law gains (*K*_*θ*_, *K*_*δ*_). The sliding surface gains should be positive to ensure the asymptotic decay of the error. The switching law gains are chosen to ensure the robustness properties of the controller. High controller gains may ensure superior robustness; however, it will require high control effort to swiftly attain stability. This high gain not only causes oscillations due to the inherent phase lag in the system but also drops the energy efficiency of the system. The robustness of the controller is also linked to the availability of accurate model information. If the model is accurately known and is subjected to zero disturbances, then the small controller gains will serve the purpose in both nominal and robust scenarios. However, in real-world scenarios, the model information cannot represent the physical system with absolute accuracy. That calls for the design of SMC with high controller gains to achieve robustness characteristics. In the proposed work, we have ensured the accurate availability of modeling information by pinpointing the parameters that are more vulnerable to the changes. These parameters are estimated online and provided to the controller. Consequently, we have ensured the small gains to achieve robust and nominal performance. The gains are heuristically chosen to best fit the closed-loop performance of the system.

The second type of parameter refers to the tuning variables of the Extended Kalman Filter. Three tuning parameters are required to carefully tune in Kalman filter estimation algorithms. They are *P*_0_, *Q*, and *R*. *P*_0_ is the initial state error covariance matrix having only diagonal entries that refers to the initial error of system states $\theta_{P},{\dot{\theta }}_{P},x_{wm},{\dot{x}}_{wm},\delta ,\dot{\delta },M_{R},L_{r}$. The pitch angle, pitch rate, yaw angle, yaw rate, forward distance traveled, and forward velocity are measured using the IMU package. The possible initial error due to the measurements of IMU is considered here. Moreover, the possible initial error of mass of the rider and its length are considered ±30% of the nominal values. The parameter *Q* refers to the state covariance matrix. Its values correspond to the modeling errors in the dynamic equations. The modeling errors in the dynamic model are minor, however, the rate of change of rider mass and its length is assumed to be zero because it will not vary during the ride of Segway. It will only be uncertain at the start of the ride. The modeling deficiency of the rider’s mass and length is accommodated by selecting high gains of the corresponding entries of the *Q* matrix. The tuning of the measurement matrix *R* is done by the possible measurement errors of IMU.

### Controller stability analysis

Using the SBR model given in the Eqs (54) and (60), the sliding surfaces defined in Eqs (61) and (62), the controllers defined in Eqs (69) and (70), and the EKF estimator designed for estimating ${\hat{M}}_{R}$ and ${\hat{L}}_{r}$ will stabilize the closed-loop system. The two control laws will perform the upright stability task and tracking of yaw command, respectively. That follows with the errors defined in Eqs (59) and (60) asymptotically approaching zero.

Selecting the Lyapunov function given in Eq (72).


$$V=\frac{1}{2}{S^{2}}_{\theta }+\frac{1}{2}{S^{2}}_{\delta }>0$$ (72)

Differentiating Eq (72) and expanding its terms yields Eq (73).


$$\dot{V}=S_{\theta }({\ddot{\theta }}_{Pe}+a_{\theta }{\dot{\theta }}_{Pe})+S_{\delta }({\ddot{\delta }}_{e}+a_{\delta }{\dot{\delta }}_{e})$$ (73)

Taking the time derivative of Eqs (59) and (60) twice and substituting the resultant expression in Eq (73) we will get Eq (74)

$$\dot{V}=S_{\theta }({\ddot{\theta }}_{P}-{\ddot{\theta }}_{Pd}+a_{\theta }{\dot{\theta }}_{Pe})+S_{\delta }(\ddot{\delta }-{\ddot{\delta }}_{d}+a_{\delta }{\dot{\delta }}_{e})$$ (74)

Substituting Eqs (52), (58), (69), and (70) in Eq (74) and simplifying leads to Eq (75).


$$\dot{V}=\frac{S_{\theta }[(C_{1}{\hat{B}}_{1}-{\hat{C}}_{1}B_{1})+{\ddot{\theta }}_{Pd}(B_{1}\hat{A}-A{\hat{B}}_{1})+a_{\theta }{\dot{\theta }}_{Pe}({\hat{B}}_{1}A-\hat{A}B_{1})-\hat{A}B_{1}K_{\theta }\tanh\left(S_{\theta })\right]}{A{\hat{B}}_{1}}+\frac{S_{\delta }[(A_{3}-{\hat{A}}_{3})a_{\delta }{\dot{\delta }}_{e}+({\hat{A}}_{3}-A_{3}){\ddot{\delta }}_{d}-K_{\delta }\tanh\left(S_{\delta }\right){\hat{A}}_{3}]}{A_{3}}$$ (75)

**Fig 16:**
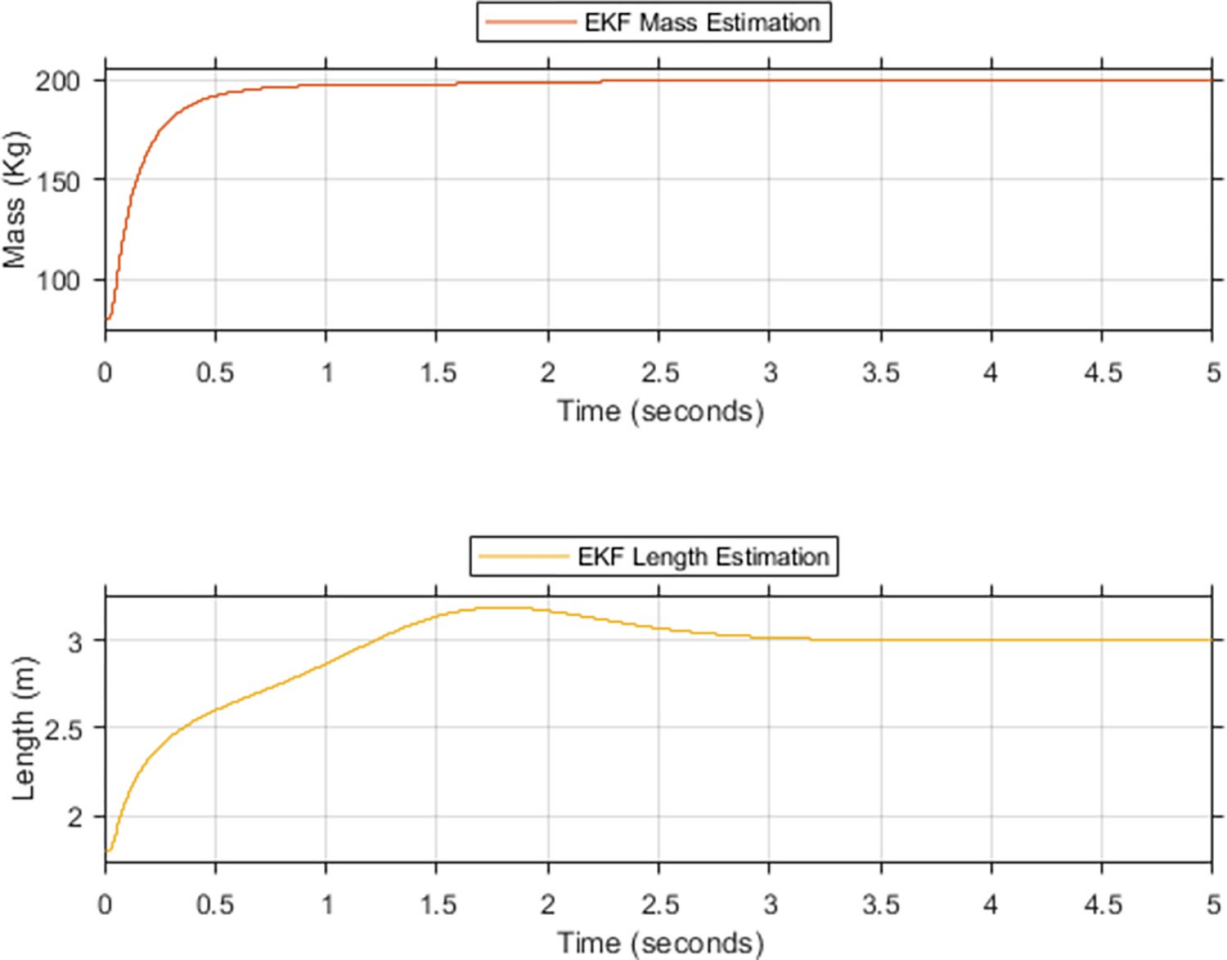
EKF based estimation for the upper limit of (a) rider’s mass, (b) rider’s length.

**Fig 17:**
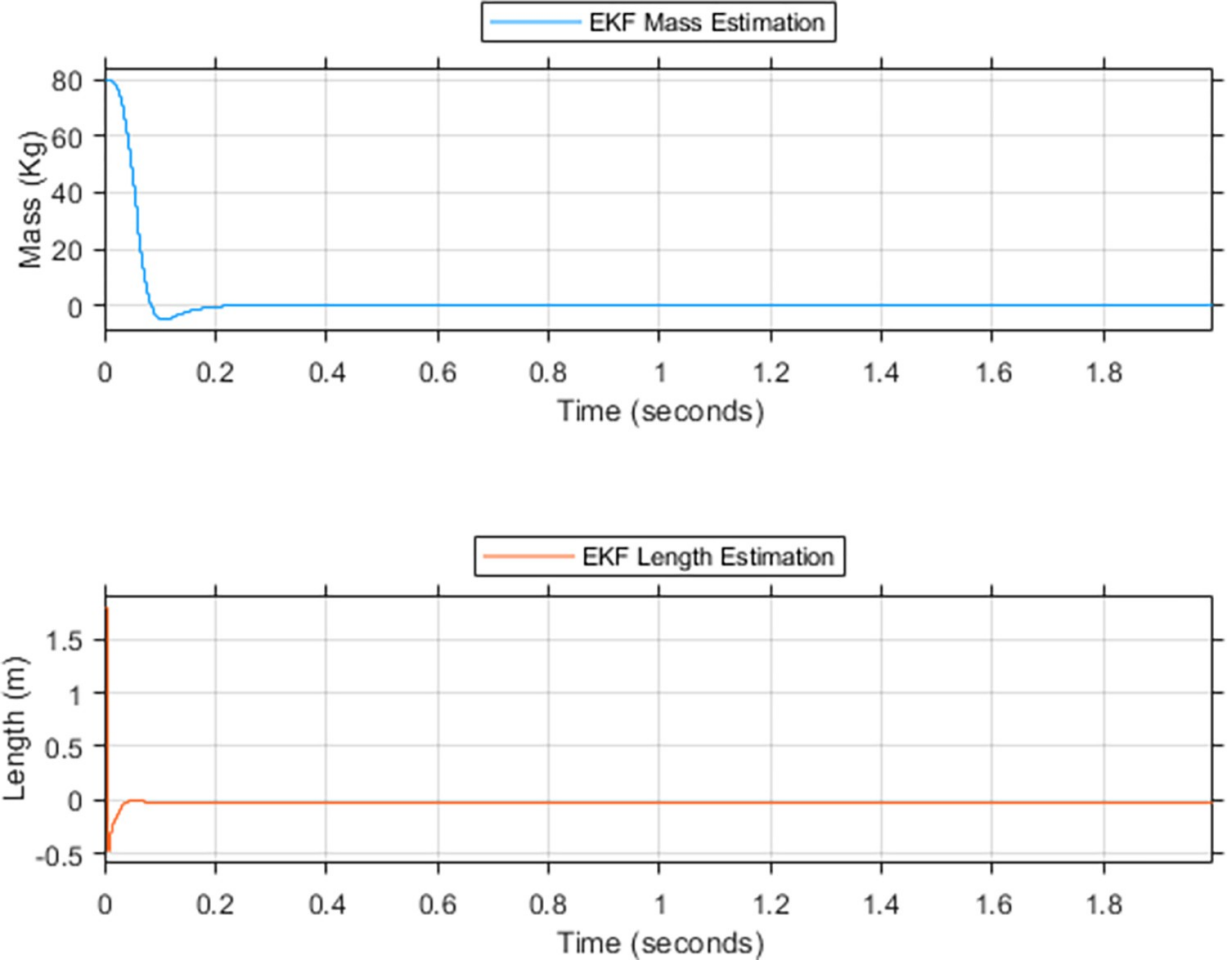
EKF rider’s estimation for lower limit (a) mass (b) length.

Figs 16 and 17 depict the convergence of ${\hat{M}}_{R}$ and ${\hat{L}}_{r}$ to ***M***_***R***_ and ***L***_***r***_, respectively. This convergence leads to the equation of following terms. $A=\hat{A},\;B_{1}={\hat{B}}_{1},\;C_{1}={\hat{C}}_{1}$, and $A_{3}={\hat{A}}_{3}$. Taking into account these equations, Eq (75) can be rewritten as Eq (76).


$$\dot{V}={-(S}_{\theta }K_{\theta }\;\tanh\left(S_{\theta })+S_{\delta }K_{\delta }\;\tanh\left(S_{\delta }\right)\right)<0$$ (76)

### Remark

Hence, The ESMC controller ensures the Lyapunov stability of the SBR defined by Eqs (54) and (60). The controller ensures that the errors defined in Eqs (59) and (60) asymptotically converge to zero within a finite time. In the proposed method, EKF made an estimate of ${\hat{M}}_{R}$ and ${\hat{L}}_{r}$ and provides that information to the SMC. The method ensures the closed-loop stability of the system. The proposed method also mitigates the chattering issues that usually come due to the selection of large controller gains for robustness in the conventional SMC.

The performance of the proposed ESMC control scheme is evaluated using rigorous simulations for different cases.

### Results and discussion

Simulations are conducted to validate the proposed ESMC balancing control. System parameters used for this purpose are listed in the nomenclature and acronyms section. The objective of these simulations is to demonstrate the enhanced performance of the proposed method on account of the estimations made for ${\hat{M}}_{R}$ and ${\hat{L}}_{r}$. This includes the illustration of improved performance at low *K*_*θ*_ gain. Ultimately leading to reduced initial magnitude and oscillations in the control effort. Therefore, maximizing the performance of ESMC with a minimum control effort requirement. The simulation scenarios emulate 7 cases to effectively depict the performance comparison between ESMC and SMC systems. Cases 1–4 consider the ‘upper limit’ of the payload with *M*_*R*_ = 200 kg and *L*_*r*_ = 3 *m*. It starts with the comparison of ESMC and SMC at similar gains for *K*_*θ*_. Progressively, the gain *K*_*θ*_ increases, effectuating the performance of the SMC system to approach the ESMC system. Finally, a combination of low *K*_*θ*_ gain for ESMC and high *K*_*θ*_ gain for SMC is used in such a way that the performance of the SMC based system becomes better than the ESMC based system. The control efforts of the two systems are then compared to justify the proposed solution. Cases 5–7 consider the ‘lower limit’ of the payload with *M*_*R*_ = 0 kg and *L*_*r*_ = 0 *m*. To justify the solution at the lower limit, a similar progressive scheme is used as in cases 1–4. For the upper and lower limits discussed in these cases, EKF is used for estimation. Fig 16 shows the simulation results of EKF estimates for the upper limit of unknown parameters. This is done using the proposed algorithm discussed in the previous section. Initial values that initiate the iterative estimation process are taken as the nominal values of the system parameters. For *M*_*R*_ and *L*_*r*_, they are taken as 80 kg and 1.8 meters, respectively. Performance of the proposed technique is tested under extreme payload variations. For mass and length, variations in addition to the ones caused by the dynamics of the rider are incorporated e.g. the mass and length of something being carried by the rider. EKF converges to 96% and 86.6% of the true value of the rider’s mass and length respectively in 0.5 seconds. It takes 3.3 seconds for the EKF to achieve 100% convergence. There is an overshoot of 6% in the estimation of the rider’s length. This is due to the relatively low quantitative variation of the length from the nominal value as compared to the mass. Fig 17 shows the simulation results for the lower limit. It takes 0.28 seconds for the EKF to achieve 100% convergence. Due to low inertia of the system, EKF converges relatively quickly. This speed comes with an undershoot that is settled instantly. These estimation results are fed to the SMC and the performance comparison of ESMC and SMC based systems is made for the 7 cases.

### Case 1: Comparison between ESMC and SMC for gains *K*_*θ*_ = 2, *a*_*θ*_ = 1, *K*_*δ*_ = 1, *a*_*δ*_ = 1 and upper limit of *M*_*R*_, *L*_*r*_

**Fig 18:**
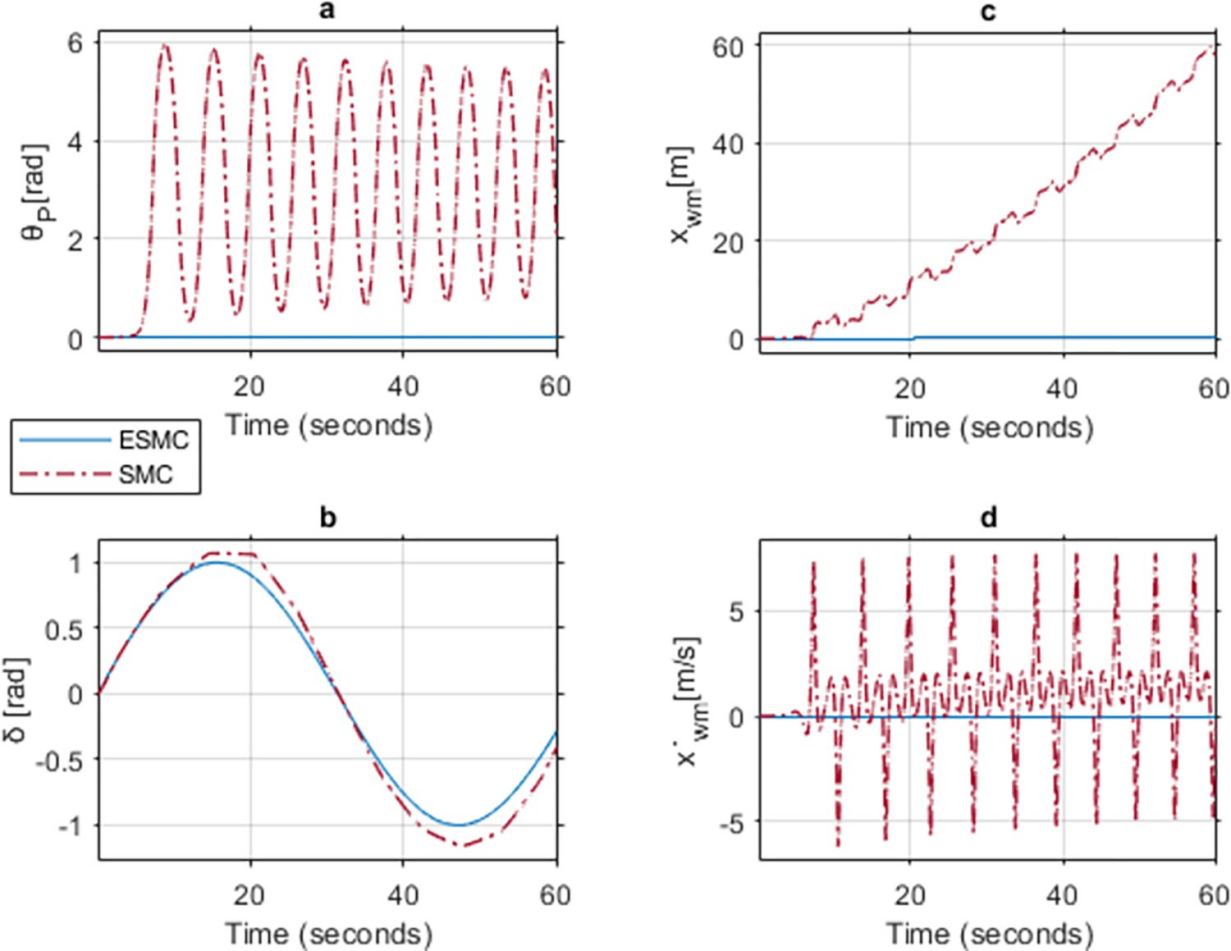
A comparison of system response for case 1: (a) Pitch angle, (b) Yaw angle, (c) Distance, (d) Velocity.

Initially, the Theta controller gain *K*_*θ*_ is taken as 2 for both ESMC and SMC based systems. The controller tuning variable for error convergence *a*_*θ*_ is taken as 1. As our main concern is pitch angle stability, the gains for *K*_*δ*_ and *a*_*δ*_ are taken as 1 throughout the case study. Fig 18 shows a comparison of system response of the two systems for the states: pitch angle (*θ*_*P*_), yaw angle (*δ*), distance (*x*_*wm*_), and velocity (${\dot{x}}_{wm}$). At this gain, SMC based system becomes unstable, but the ESMC manages to stabilize. For the proposed ESMC based system, *θ*_*P*_ and *δ* meet the desired angle demand. It also maintains a relatively constant position with negligible velocity. In the SMC based system, however, *θ*_*P*_ starts oscillating. This jerky motion causes the position of the vehicle to change with an oscillating velocity. Also, *δ* deviates from its desired trajectory.

### Case 2: Comparison between ESMC and SMC for gains *K*_*θ*_ = 6, *a*_*θ*_ = 1, *K*_*δ*_ = 1, *a*_*δ*_ = 1 and upper limit of *M*_*R*_, *L*_*r*_

**Fig 19:**
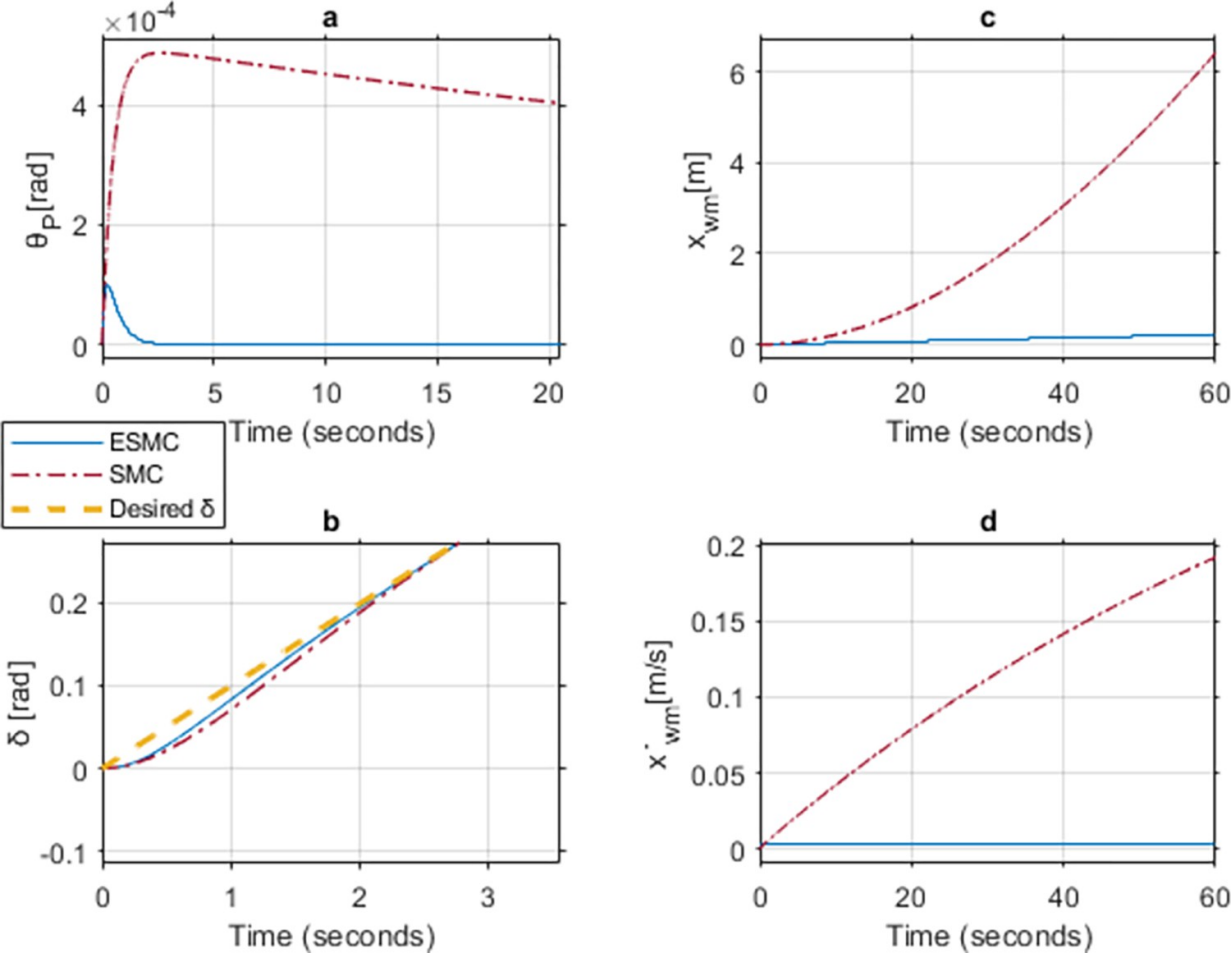
A comparison of system response for case 2: (a) Pitch angle, (b) Yaw angle, (c) Distance, (d) Velocity.

Fig 19 shows the performance comparison at *K*_*θ*_ = 6 for the two systems. Unlike case-1, the SMC based system stabilizes at this gain. This is the minimum control gain at which the SMC based system becomes stable. Although, *θ*_*P*_ deviation is extremely small for the SMC based system, however, it fails to reach the desired angle value even after 20 seconds. To ensure system stability, the velocity of the vehicle increases and so does the distance covered. This delay causes the system to reach a high velocity by the time *θ*_*P*_ becomes zero. On the other hand, the ESMC based system stabilizes within 2 seconds with negligible velocity and distance covered. *δ* tracks the desired angle faster with the ESMC based system with the max difference of 0.012 rad between the two systems.

Table 1 shows the maximum difference between the states of the two systems with ESMC based system leading in performance.

**Table 1:** Maximum difference between the states of ESMC and SMC for Case 2.

State	Difference	Unit
Max *θ*_*P*_ difference	0.0005	rad
Max *δ* difference	0.012	rad
Difference in *x*_*wm*_ after 1 min	6.2	m
Max difference in velocity ${\dot{x}}_{wm}$	0.18	m/s

### Case 3: Comparison between ESMC and SMC for gains *K*_*θ*_ = 50, *a*_*θ*_ = 1, *K*_*δ*_ = 1, *a*_*δ*_ = 1 and upper limit *M*_*R*_, *L*_*r*_

**Fig 20:**
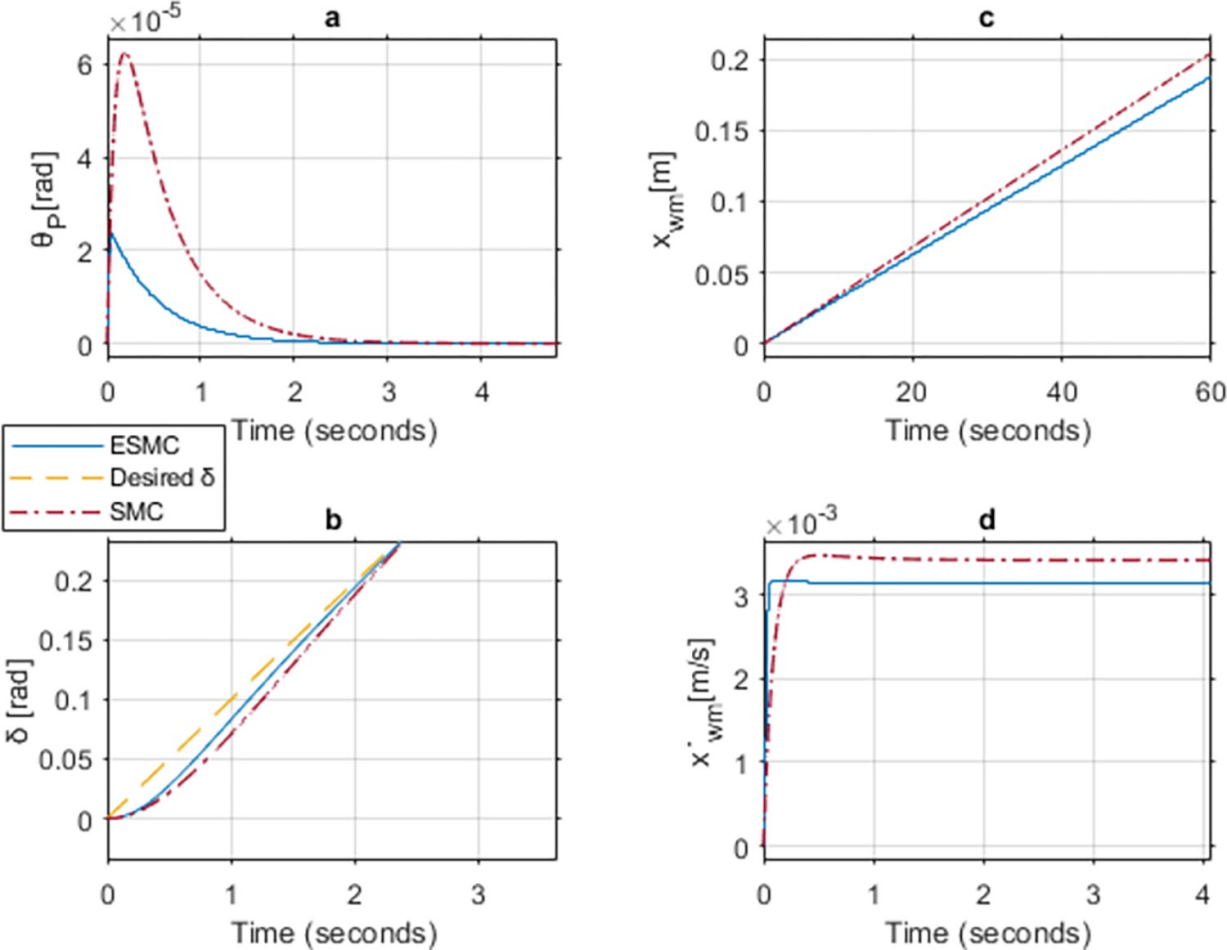
A comparison of system response for case 3: (a) Pitch angle, (b) Yaw angle, (c) Distance, (d) Velocity.

Fig 20 shows simulation results for case 3. For both the systems, the controller gain *K*_*θ*_ is taken as 50. With the progressive increase in controller gain, performance of both SMC and ESMC tend to approach one another. This is because, at such high gain, SMC becomes robust enough to tackle the uncertainties that previously were affecting the system’s performance. However, the proposed method of ESMC based system still outperforms the SMC based system. This shows the effectiveness of estimation even at high controller gains. *δ* still tracks the desired angle faster with the ESMC based system with the max difference of 0.012 rad between the two systems.

Table 2 shows the maximum difference between the states of the two systems with ESMC based system performing slightly better than the SMC based system.

**Table 2:** Maximum difference between the states of ESMC and SMC for Case 3.

State	Difference	Unit
Max *θ*_*P*_ difference	4.36e-5	rad
Max *δ* difference	0.012	rad
Difference in *x*_*wm*_ after 1 min	0.016	m
Max difference in velocity ${\dot{x}}_{wm}$	0.0015	m/s

### Case 4: Comparison between ESMC having gains *K*_*θ*_ = 2, *a*_*θ*_ = 1, *K*_*δ*_ = 1, *a*_*δ*_ = 1 and SMC having gains *K*_*θ*_ =50, *a*_*θ*_ = 1, *K*_*δ*_ = 1, *a*_*δ*_ = 1 and upper limit *M*_*R*_, *L*_*r*_

**Fig 21:**
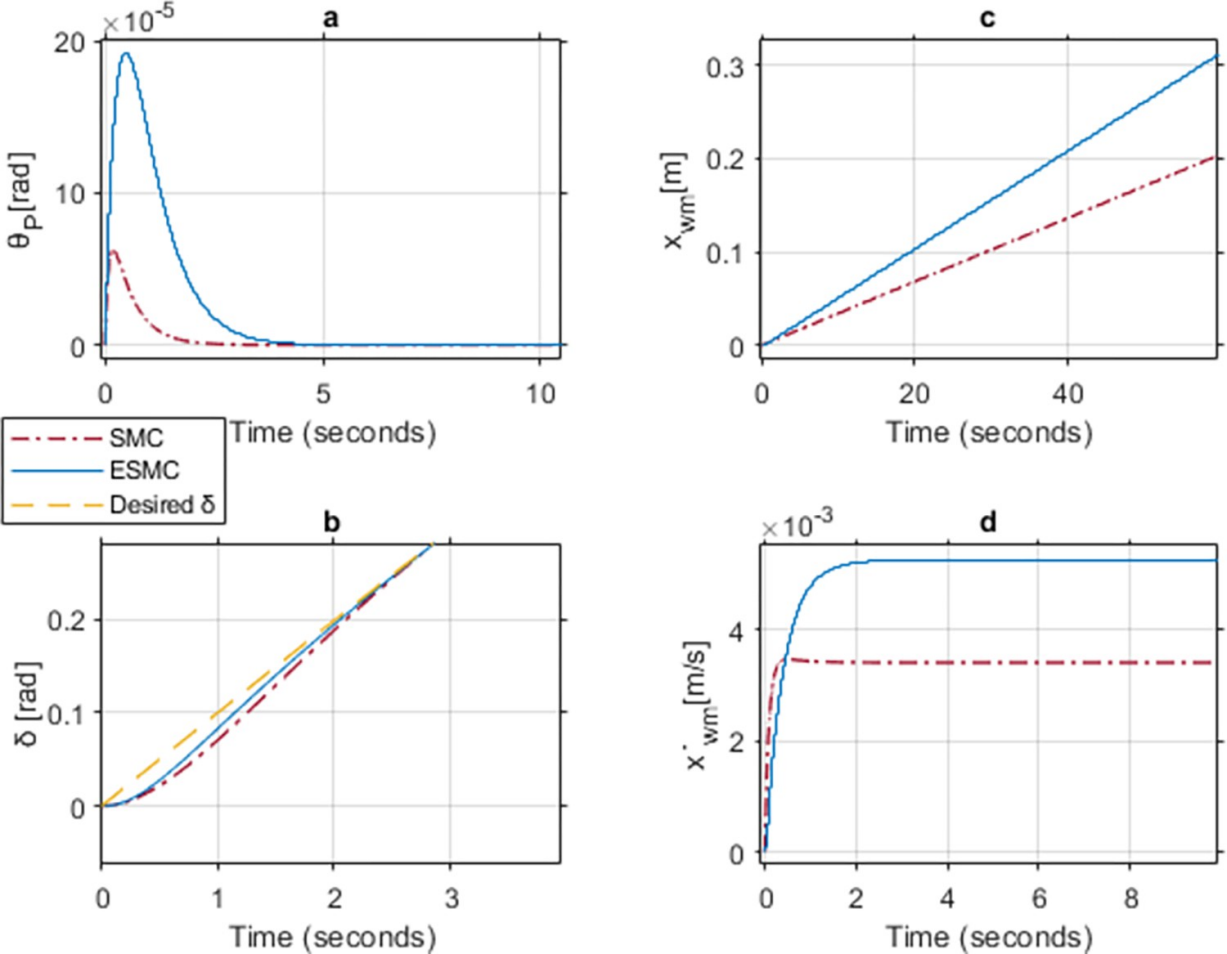
A comparison of system response for case 4: (a) Pitch angle, (b) Yaw angle, (c) Distance, (d) Velocity.

**Fig 22:**
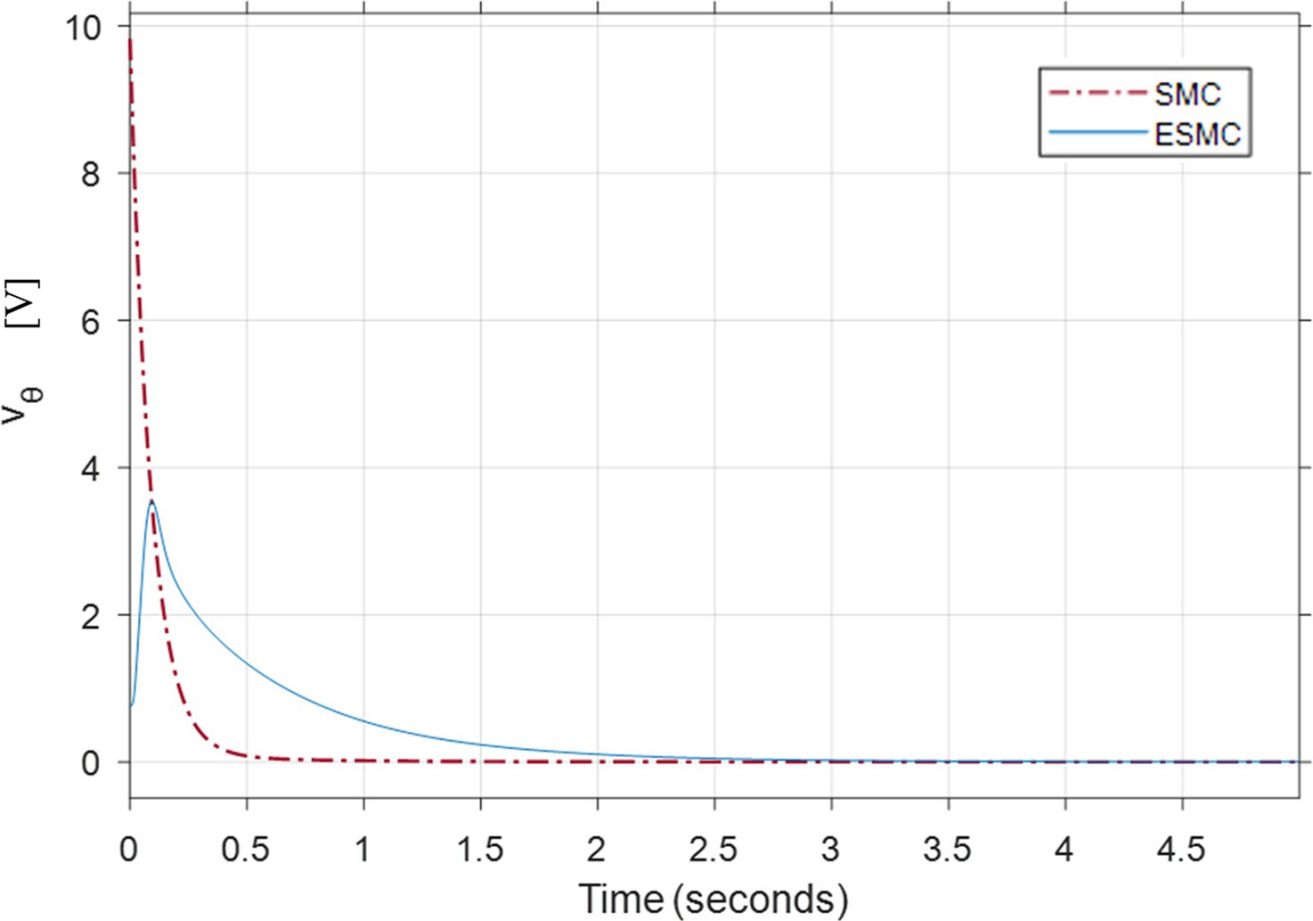
Comparison of the control effort generated by the controllers to stabilize the pitch angle.

Figs 21, 22 show the performance and control effort results for case 4. It is evident from the previous cases that increased *K*_*θ*_ gain improves the performance of the SMC based system. Hence, in this case, a comparison between high *K*_*θ*_ gain SMC based system and low *K*_*θ*_ gain ESMC based system is examined. Although, this combination shows that the system with SMC gives a better performance, its high gain demands a very high initial control effort of 9.8 volts to stabilize the underactuated system. That can cause problems in a system with low saturation voltage electric motors. It also affects the energy efficiency of the system. Alternatively, the ESMC based system estimates the values of the rider’s mass *M*_*R*_ and length *L*_*r*_. This estimation enables the system to operate with a low *K*_*θ*_ gain of 2. This reduces the initial voltage spike in the control effort to just 0.8 volts. *δ* tracks the desired angle faster with the ESMC based system still. The max difference between the *δ* angle of the two systems is of 0.012 rad. This demonstrates the use of estimation and the effectiveness of the proposed method when *M*_*R*_ = 200 *kg* and *L*_*r*_ = 3 *m*.

Table 3 shows the maximum difference between the states and the control effort of the two systems where SMC based system’s performance is better than the ESMC based system except for *δ* angle. Control effort response of the system with ESMC is better.

**Table 3:** Maximum difference between the states of ESMC and SMC for Case 4.

State / Input	Difference	Unit
Max *θ*_*P*_ difference	0.000148	rad
Max *δ* difference	0.012	rad
Difference in *x*_*wm*_ after 1 min	0.1	m
Max difference in velocity ${\dot{x}}_{wm}$	0.001	m/s
Initial *v*_*θ*_ difference	9	V

### Case 5: Comparison between ESMC and SMC for gains *K*_*θ*_ = 6, *a*_*θ*_ = 1, *K*_*δ*_ = 1, *a*_*δ*_ = 1 and lower limit *M*_*R*_, *L*_*r*_

**Fig 23:**
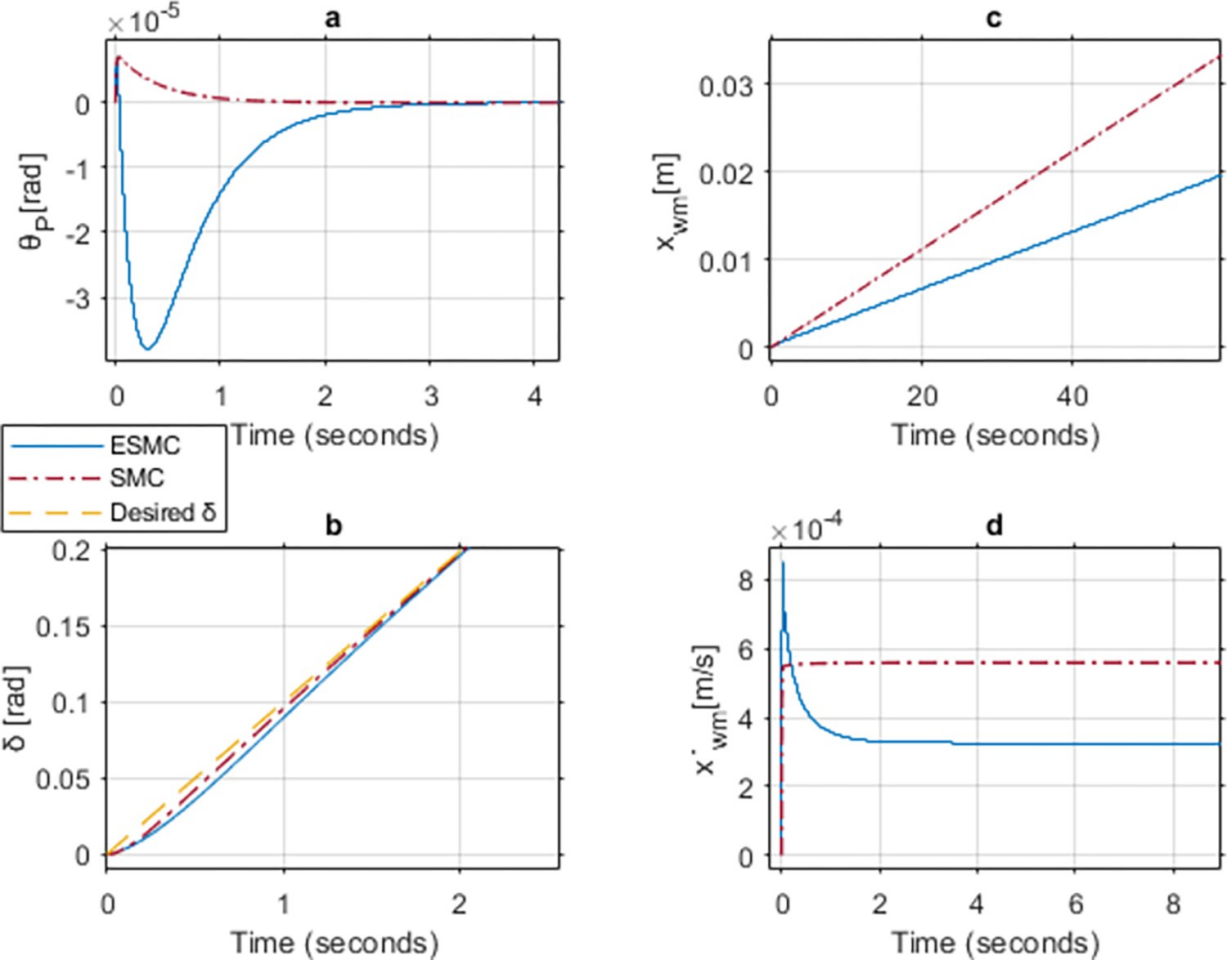
A comparison of system response for case 5: (a) Pitch angle, (b) Yaw angle, (c) Distance, (d) Velocity.

With the conclusion of the upper limit, the simulation is turned towards the lower limit of the payload. This scenario indicates that there is no rider or payload. Fig 23 shows the results of case 5. Due to the absence of payload, the system has a lower moment of inertia. Hence, its stabilization is relatively easy. Apparently, *θ*_*P*_ stabilizes more effectively with the SMC based system but ends up generating much more velocity in comparison to the ESMC based system. This increased velocity further deviates the position of the vehicle. This happens because the system falls backward initially due to its low inertia. The ESMC based system is aware of this response and does not over actuate the system. The initial velocity spike brings *θ*_*P*_ back to zero from negative. Hence, reducing the net velocity due to cancellation of the positive velocity effect with *θ*_*P*_ stabilization. This in return leads to a smaller displacement from the starting position at a given point in time. *δ* tracks the desired angle slightly better with the SMC based system for the initial few seconds. After reaching the sliding surface, both systems give the same performance for *δ* tracking.

Table 4 shows the maximum difference between the states of the two systems. *θ*_*P*_ stability and *δ* tracking response are better for the SMC based system. Whereas distance and velocity responses are better for the ESMC based system.

**Table 4:** Maximum difference between the states of ESMC and SMC for Case 5.

State	Difference	Unit
Max *θ*_*P*_ difference	3.5e-5	rad
Max *δ* difference	0.007	rad
Difference in *x*_*wm*_ after 1 min	0.01	m
Max difference in velocity ${\dot{x}}_{wm}$	0.00023	m/s

### Case 6: Comparison between ESMC and SMC for gains *K*_*θ*_ = 50, *a*_*θ*_ = 1, *K*_*δ*_ = 1, *a*_*δ*_ = 1 and lower limit *M*_*R*_, *L*_*r*_

**Fig 24:**
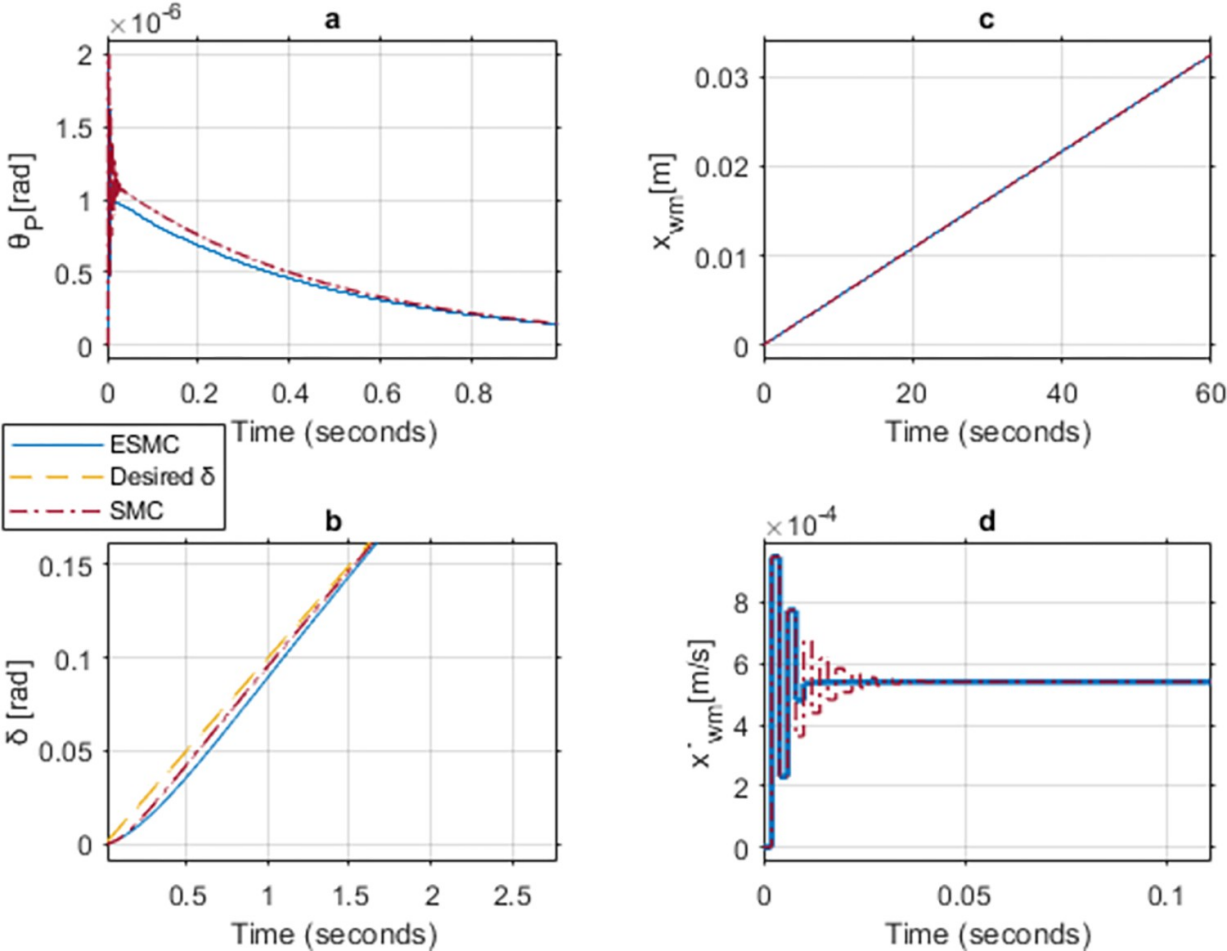
A comparison of system response for case 6: (a) Pitch angle, (b) Yaw angle, (c) Distance, (d) Velocity.

**Fig 25:**
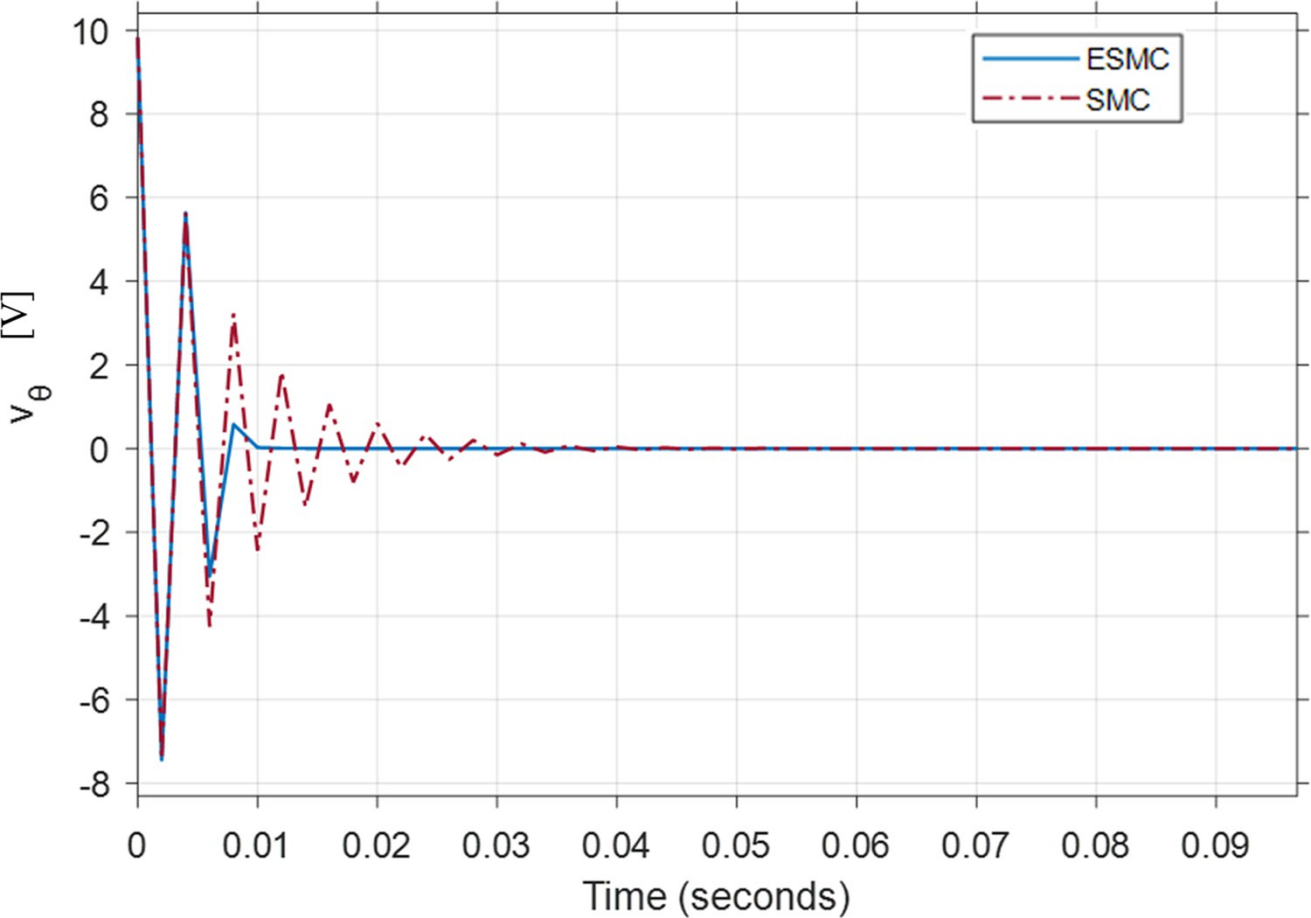
Comparison of the control effort generated by the controllers to stabilize the pitch angle.

Figs 24, 25 show the simulation results of case 5 implementing a strategy similar to the upper limit cases. Increase in *K*_*θ*_ gain to 50 results in approximately similar performance outcome for both of the systems. However, the controller without EKF tends to experience chattering in the control effort for a longer period as compared to the one with EKF. Furthermore, *θ*_*P*_, ${\dot{x}}_{wm}$, and *v*_*θ*_ response oscillates initially for both of the systems. This happens due to the high gains used in a low inertial system with no payload. These oscillations come to a halt after 0.01 seconds in the ESMC based system and 0.04 seconds in the SMC based system. The position response of both systems is the same. *δ* tracks the desired angle slightly better with the SMC based system for the initial few seconds. After reaching the sliding surface, both systems give the same performance for *δ* tracking.

Table 5 shows the maximum difference between the states and the control effort of the two systems. *θ*_*P*_ stability and velocity response are better for the ESMC based system. *δ* tracking response is better for the SMC based system whereas distance response is the same for both systems. Control effort *v*_*θ*_ gives a better response with ESMC based system.

**Table 5:** Maximum difference between the states of ESMC and SMC for Case 6.

State / Input	Difference	Unit
Max *θ*_*P*_ difference	9.6e-8	rad
Max *δ* difference	0.007	rad
Difference in *x*_*wm*_ after 1 min	1.6e-5	m
Max difference in velocity ${\dot{x}}_{wm}$	0.0001	m/s
*v*_*θ*_ difference at 0.01 sec	2.5	V

### Case 7: Comparison between ESMC having gains *K*_*θ*_ = 2, *a*_*θ*_ = 1, *K*_*δ*_ = 1, *a*_*δ*_ = 1 and SMC having gains *K*_*θ*_ = 50, *a*_*θ*_ = 1, *K*_*δ*_ = 1, *a*_*δ*_ = 1 and lower limit *M*_*R*_, *L*_*r*_

**Fig 26:**
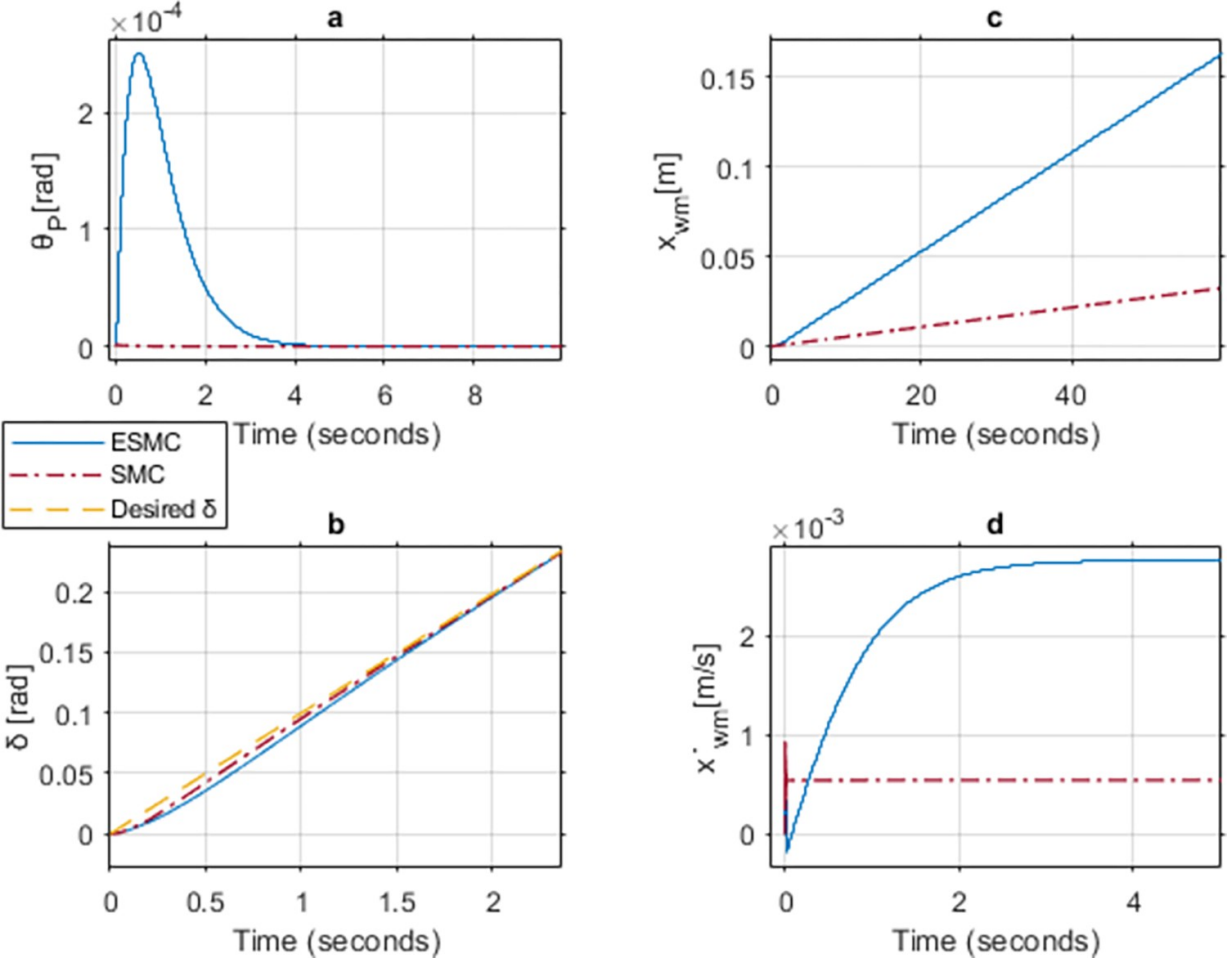
A comparison of system response for case 7: (a) Pitch angle, (b) Yaw angle, (c) Distance, (d) Velocity.

**Fig 27:**
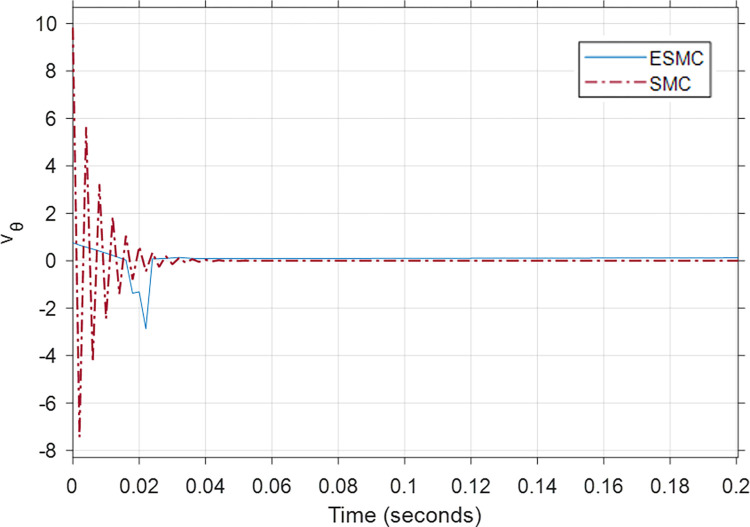
Comparison of the control effort generated by the controllers to stabilize the pitch angle.

Figs 26, 27 show the performance and control effort *v*_*θ*_ response for case 7. Similar to case 4, in this comparison, the performance of the systems is observed under a low *K*_*θ*_ gain for ESMC based system and a high *K*_*θ*_ gain for the SMC one. As expected, the SMC based high *K*_*θ*_ gain, system performs considerably better when it comes to system states. Apart from the initial oscillations in *θ*_*P*_ and the system velocity, the pitch angle deviates 0.00017 rad less from the desired *θ*_*P*_ in the SMC based system. After 1 minute, the SMC based system face 0.13 meter less displacement from the starting point as compared to the ESMC one. This is due to the difference in the velocity between the two which is 0.002 m/s. *δ* also slightly performs better in the SMC based system. However, the initial control command for the system with ESMC is 0.8 V as compared to 9.8 V for the system with SMC. Additionally, the control command of the system with SMC is subjected to more chattering. These results conclude that a system with low controller gain in combination with the estimation of unknown payload parameters work better than a system with high controller gain without any estimation when there is no rider or payload.

This study considers the length (Lr) and mass (Mr) of the rider as the parameters of interest. Variations in these parameters affect the rider’s moment of inertia (Jr) and, consequently, the system’s moment of inertia as described in Eq 26 and 24, respectively. Additionally, changes in these parameters affect the vehicle’s center of gravity (L), but the effect is relatively low compared to the changes in the rider’s moment of inertia.

**Fig 28:**
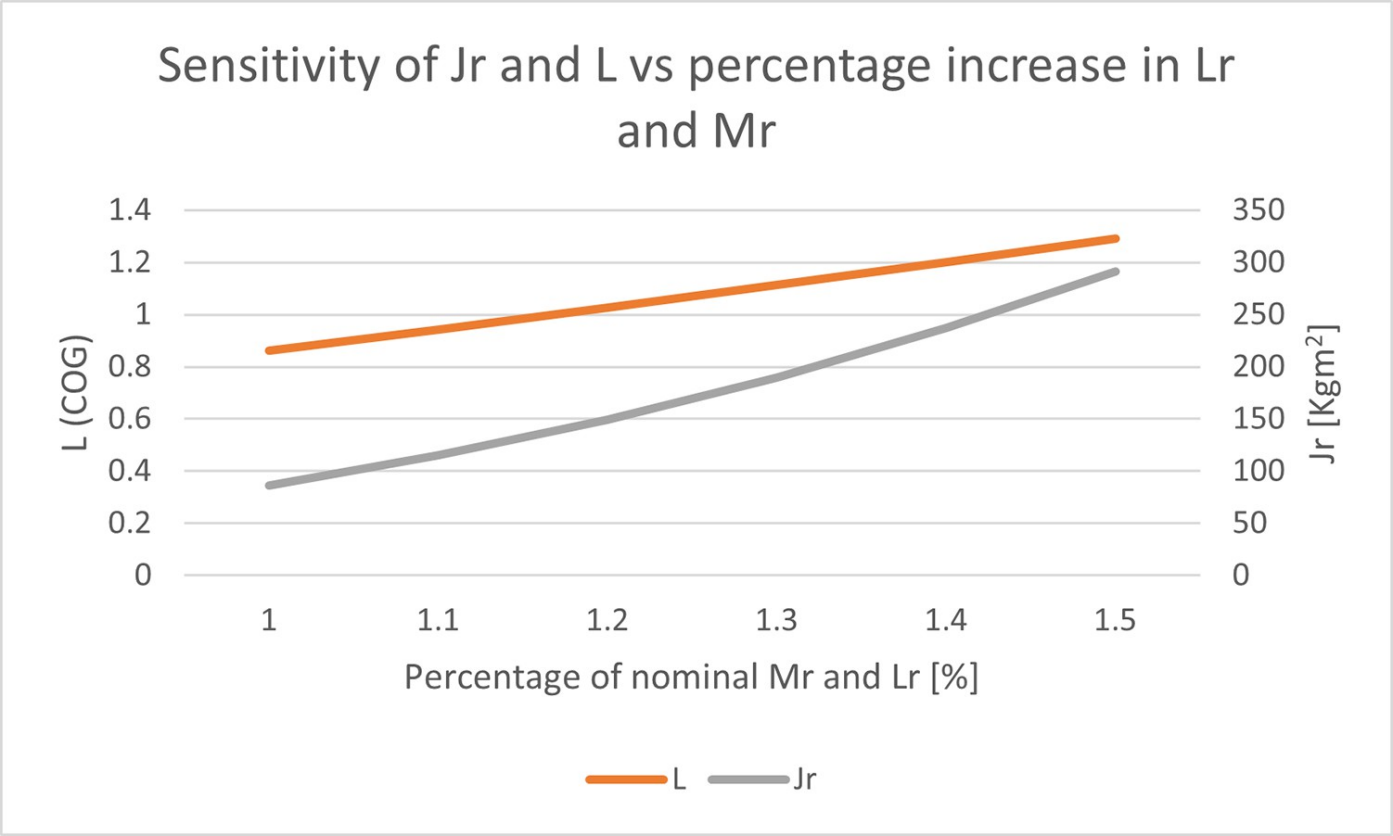
Changes in L and Jr resulting from an incremental 10% increase in Lr and Mr from their nominal values of 1.8 meters and 80 kg, respectively.

**Fig 29:**
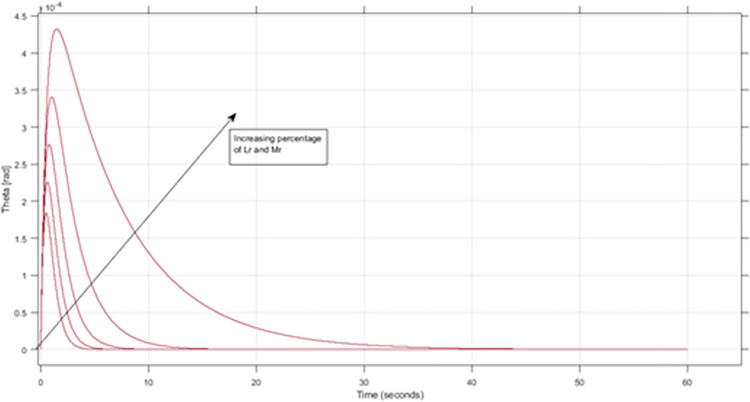
The sensitivity of pitch angle resulting from a similar increase in the values of Lr and Mr.

Fig 28 illustrates the changes in L and Jr resulting from an incremental 10% increase in Lr and Mr from their nominal values of 1.8 meters and 80 kg, respectively. Fig 29 depicts the sensitivity of pitch angle resulting from a similar increase in the values of Lr and Mr. The system becomes unstable with low controller gains when Mr is 150% of its nominal value (120kg) and Lr is 2.7. However, the proposed method can keep the system stable up to 250% of the nominal value of Mr with the same controller gains. The detail analysis is performed in results and discussion section where in section 6.1, performance of both controllers under small gains and maximum change in rider length and mass is conducted. In Fig 18 of manuscript, it can be seen that SMC controller with small controller gain is not able to maintain stability while the proposed method effectively stabilizes the system. Overall, the results of this study highlight the importance of considering the rider’s length and mass as crucial parameters that affect the stability of self-balancing vehicles.

Table 6 shows the maximum difference between the states and the control effort response of the two systems. State response performance of the SMC based system is better, yet the control effort response strengthens the utility of the proposed method.

**Table 6:** Maximum difference between the states of ESMC and SMC for Case 7.

State / Input	Difference	Unit
Max *θ*_*P*_ difference	0.00017	rad
Max *δ* difference	0.007	rad
Difference in *x*_*wm*_ after 1 min	0.13	m
Max difference in velocity ${\dot{x}}_{wm}$	0.002	m/s
Initial *v*_*θ*_ difference	9	V

### Validation and comparison of proposed control algorithm

The performance of the proposed ESMC technique is compared with the earlier techniques in the literature to justify its novelty. Table 7 shows that Butler and Bright proposed a control technique using the linear quadratic regulator. They took into account the variation in payload but the maximum load considered was 70 kg as opposed to the 200 kg considered in the proposed ESMC technique. Additionally, the control response was very sluggish with a settling time of 10 seconds for the tilt angle, and no information about the control torque was provided. Shui-Chun Lin achieved stability using the linear adaptive robust controller. In these experiments, author incorporated the variation of payload but with a maximum of only 85 kg. Although, the settling time of the tilt angle was satisfactory, it experienced an undershoot of 5e-3 rad. No information regarding the control effort was provided for this case either. Zhao-Qin Guo used a sliding mode controller in combination with an observer to achieve robust control. Even though the study considered a static payload of just 1.6 kg, the controller response was sluggish with a settling time of 8 seconds and an undershoot of 0.02 rad. Moreover, oscillations of 0.05 units can be noted in the tilt angle and control effort. Nguyen Ngoc Son utilized an adaptive backstepping control technique to realize the stability of the SBR. Although, the controller response was quick with a settling time of 0.4 seconds, it only considered a static payload of 26 kg. The maximum control effort required for stability was a soaring 200 Nm in contrast to the 33 Nm requirement in the proposed ESMC technique. Byung Woo Kim examined an SBR with a dynamic load with a maximum of 85 kg using a backstepping controller with unknown control coefficients and model uncertainties. It took 3 seconds for the tilt angle to settle with an undershoot of 0.17 rad. The maximum required control effort, however, rocketed to 150 Nm with oscillations of ± 50 Nm. Nasim Esmaeili proposed a backstepping controller in combination with a two-layered SMC. This technique achieved upright stability within 1 second but experienced oscillations of magnitude ± 0.1 rad. Furthermore, it considered a 0.38 kg static payload, and the initial control torque requirement for stability was 4 V with an oscillation of ± 3 V. Ji-Hyun Park presented a hardware solution to the stability control problem by using a control moment gyroscope. An observer was used to estimate the disturbances and eventually actuate the control moment gyroscope. Stability control was achieved using a linear quadratic regulator. Tilt angle settled in around 1.5 seconds but experienced an oscillation of ± 0.02 rad. Additionally, the information regarding the control effort was not discussed. Finally, Thomas Johnson realized a perceptual control technique on a static payload of 0.5 kg. Even though it rejected disturbances effectively, the stability response was 1.5 seconds slower than the proposed technique with an undershoot, noticeable oscillations, and a steady-state error. The proposed technique can handle payloads up to 200kg with the maximum and initial required control effort of 3.3 V and 0.8 V, respectively, with no oscillation. The tilt angle settled in about 3.5 seconds with no steady-state error or oscillations.

**Table 7:** Comparison of the proposed ESMC technique with the earlier work reported in the literature.

Techniques (Year)	Payload (kg)	Controller	Observer	Estimation Parameter	Tilt Angle (rad)						Control Effort (V/Nm)		
					Initial	Max Defl.	SS error	ST (sec)	Undershoot (rad)	Osci. Mag. (rad)	Initial	Maximum	Oscillation
**Butler and Bright** 2010	Dynamic with max 70 Kg	LQR	-	-	0	0.02	-	10	0.02	-	-	-	-
**Shui-Chun Lin** 2011	Dynamic with max 85 Kg	Linear Adaptive Robust Controllers	-	-	0.3	-	-	0.3	5e-3	-	-	-	-
Zhao-Qin Guo 2014	Static 1.6 kg	SMC	Sliding mode observer	Tire Joint Friction, Slope Angle	0.1	-	-	8	0.02	± 0.05	0.06 V	0.06 V	± 0.05 V
**Nguyen Ngoc Son** 2014	Static 26 Kg	Adaptive Backstepping Controller	-	-	0.15	-	-	0.4	-	-	-	200 Nm	-
**Byung Woo Kim** 2016	Dynamic with max 85 kg	Backstepping Controller with Unknown Coef. & Model Uncertainty	-	-	0.35	-	-	3	0.17	-	-	150 Nm	± 50 Nm
**Nasim Esmaeili** 2017	Static 0.38 kg	Backstepping + two-layer SMC	-	-	0	0.3	-	1	0.025	± 0.1	4 V	4 V	± 3 V
**Ji-Hyun Park** 2018	-	LQR	Disturbance Observer + Control Moment Gyroscope	Disturbance at the Center of Mass	0	-	-	1.5	-	± 0.02	-	-	-
**Thomas Johnson** 2020	Static 0.5 kg	Perceptual Control	-	-	-	-	0.035	5	0.07	± 0.035	-	-	-
**Proposed Work**	Varying with 200 kg max	Sliding Mode Controller	Extended Kalman Filter	Rider’s Height and Mass	0	1.9e-4	0	3.5	0	-	0.8 V	3.3 V	-

### Conclusions and recommendations

Most of the available solutions for the two-wheeled Self-Balancing Robot (SBR) either have constraint on the payload or their performance is compromised. In this work, a robust control with unknown payload parameters is realized, A detailed mathematical model, incorporating payload and motor dynamics, is derived and utilized to formulate the control equations for a Sliding Mode Controller (SMC). This controller along with an Extended Kalman Filter (EKF) is used to regulate the stability of the robot. Moreover, the detailed model of the SBR helps to improve the performance of the control system. EKF estimates the unknown parameters that are used as input to the SMC which in turn helps to boost the performance of the system. Rigorous simulations are carried out to validate the effectiveness of the proposed method for various scenarios using seven case studies. These simulation cases are identified according to the controller gain and payload variations. The results of the proposed method with parameter estimation (ESMC) are compared with the system without parameter estimation (SMC). It is illustrated that the performance of ESMC turns out to be better when similar control gains are used for both control systems. The combination of gains that exhibit a better performance for the SMC system, reveals a notable difference between the two systems in the initial control effort requirement for stability. The proposed technique is effective for the structured environment and indoor applications. It has a great scope for warehouses and predefined tasks for indoor environment.

For future recommendations, the system may be subjected to the outdoor harsh environment that can introduce unwanted disturbances in the system. These issues along with performance targets may be addressed in the future work. Since, the mathematical model has a provision to accommodate disturbances, a solution may be developed in future to design control system for unstructured environment and outdoor applications. This further signifies the effectiveness of the proposed ESMC technique.

### Nomenclature and acronyms

#### Constants and variables

**Table:** 

**Symbol**	**Value [Unit]**	**Parameter**
*θ*_*P*_	[rad]	Pitch angle of the robot about z-axis
${\dot{\theta }}_{P}$	[rad/s]	Pitch angular velocity about z-axis
${\ddot{\theta }}_{P}$	[rad/s^2^]	Pitch angular acceleration about z-axis
*x*_*wm*_	[m]	Position of the robot
${\dot{x}}_{wm}$	[m/s]	Velocity of the robot
${\ddot{x}}_{wm}$	[m/s^2^]	Acceleration of the robot
*x*	[m]	Position of chassis
$\dot{x}$	[m/s]	Linear velocity of chassis
$\ddot{x}$	[m/s^2^]	Linear acceleration of chassis
*δ*	[rad]	Yaw angle of the robot about y-axis
$\dot{\delta }$	[rad/s]	Yaw angular velocity about y-axis
$\ddot{\delta }$	[rad/s^2^]	Yaw angular acceleration about y-axis
*θ*_*WL*_, *θ*_*WR*_	[rad]	Pitch angle of left and right wheel
${\dot{\theta }}_{WL},{\dot{\theta }}_{WR}$	[rad/s]	Pitch angular velocity of left and right wheel
${\ddot{\theta }}_{WL},{\ddot{\theta }}_{WR}$	[rad/s^2^]	Pitch angular acceleration of left and right wheel
*x*_*WL*_, *x*_*WR*_	[m]	Sector length of left and right wheel
*y*_*WL*_, *y*_*WR*_	[m]	Vertical position of the wheel
*R*	0.2 [m]	Wheel radius
*D*	0.48 [m]	Lateral distance between the contact patches of the wheels
*g*	9.8 [m/s^2^]	Acceleration due to gravity
*f*_*dp*_	[N]	Disturbance force at COG of robot
*f*_*dl*_, *f*_*dr*_	[N]	Disturbance forces at left and right wheel
*C*_*L*_, *C*_*R*_	[Nm]	Control torques from left and right wheel
*C*_*θ*_	[Nm]	Control torque for pitch angle
*C*_*δ*_	[Nm]	Control torque for yaw angle
*t*_*m*_	[Nm]	Torque applied by motor
*t*_*a*_	[Nm]	Applied torque on motor
*H*_*TL*_, *H*_*TR*_	[N]	Horizontal reaction forces between ground and wheels
*H*_*L*_, *H*_*R*_	[N]	Horizontal reaction forces between wheels and chassis
*V*_*TL*_, *V*_*TR*_	[N]	Vertical reaction forces between ground and wheels
*V*_*L*_, *V*_*R*_	[N]	Vertical reaction forces between wheels and chassis
*M*_*W*_	3 [kg]	Mass of wheel
*M*_*R*_	80 [kg] (nominal)	Mass of rider
*M*_*V*_	30 [Kg]	Mass of chassis (robot)
*M*	[kg]	Total mass of the system
*h*	1.03 [m]	Height of chassis
*L*_*r*_	1.8 [m] (nominal)	Length of rider
*L*	[m]	Distance between z-axis and COG of chassis
*J*_*r*_	[Kgm^2^]	Rider moment of inertia
*J*_*v*_	[Kgm^2^]	Robot moment of inertia
*J*	[Kgm^2^]	Total moment of inertia of system
*J*_*δ*_	[Kgm^2^]	Moment of inertia about y-axis
*J*_*wl*_, *J*_*wr*_	[Kgm^2^]	Moment of inertia of wheels
*J*_*R*_	[Kgm^2^]	Rotor moment of inertia
*K*_*e*_	0.1 [Vs/rad]	Motor back EMF constant
*K*_*m*_	0.1 [Nm/A]	Motor torque constant
*K*_*f*_	0 [Nms/rad]	Motor frictional constant
*v*_*l*_, *v*_*r*_	[V]	Control voltage for left and right motor
*v*_*θ*_	[V]	Control voltage for pitch angle
*v*_*δ*_	[V]	Control voltage for yaw angle
*v*_*e*_	[V]	Back emf of motor
*i*	[A]	Armature current
*L*_*i*_	0 [H]	Motor inductance
*r*	1 [Ω]	Motor resistance
*K*_*θ*_	-	Theta controller gain
*a*_*θ*_	-	Theta controller tuning variable for error convergence
*K*_*δ*_	-	Delta controller gain
*a*_*δ*_	-	Delta controller tuning variable for error convergence
*θ*_*Pe*_	[rad]	Controller pitch angle estimation error
*θ*_*Pd*_	[rad]	Desired pitch angle
*δ*_*Pe*_	[rad]	Controller yaw angle estimation error
*δ*_*Pd*_	[rad]	Desired yaw angle
